# Gut Microbial Release of Ferulic Acid From Germinated Quinoa Alleviates Obesity‐Associated Cognitive Impairment by Activating Hippocampal Mitophagy Associated with PINK1/Parkin Pathway

**DOI:** 10.1002/advs.76686

**Published:** 2026-07-20

**Authors:** Yongli Lan, Wengang Zhang, Lei Wang, Shiyang Zhao, Yujie Song, Xinze Wang, Yutang Wang, Xijuan Yang, Shaobo Ma, Junlin Ge, Rui Guo, Xuebo Liu

**Affiliations:** ^1^ College of Food Science and Engineering Northwest A&F University Yangling Shaanxi China; ^2^ Academy of Agriculture and Forestry Sciences Laboratory for Research and Utilization of Qinghai Tibet Plateau Germplasm Resources Qinghai University Xining China; ^3^ Department of Clinical Nutrition Xijing Hospital Air Force Military Medical University Xi'an China

**Keywords:** ferulic acid, germinated quinoa, gut microbiota, mitophagy, *Roseburia*, synaptic plasticity

## Abstract

Obesity is a major risk factor for cognitive impairment and related neurodegenerative disorders. Whole grains are rich in polyphenols and dietary fiber, with intake associated with improvements in obesity and cognitive deficits. Gut microbial genes encode enzymes that metabolize dietary polyphenols, thereby influencing host metabolic and neurological health; however, the specific functional microbes and enzymes involved remain largely unknown. Here, we demonstrate that germinated quinoa (GQF), a polyphenol‐enriched whole‐grain intervention, alleviates high‐fat diet‐induced cognitive impairment and restores gut microbial diversity more effectively than native quinoa (QF). These benefits correlate with enriched *Roseburia* abundance and enhanced carbohydrate‐active enzyme (CAZyme) gene profiles, particularly genes encoding feruloyl esterase (FAE). GQF exerts neuroprotective effects via a microbiota‐substrate co‐dependent pattern, rather than simply relying on gut microbiota alone. Specifically, GQF selectively promotes FAE‐harboring *Roseburia hominis* and *R. intestinalis* proliferation, thereby facilitating the release of bound ferulic acid (FA) from GQF. Liberated FA promotes neuronal growth and synaptic plasticity by activating PINK1/Parkin‐dependent hippocampal mitophagy. These results underscore the indispensable role of specific gut microbes in unlocking the nutritional efficacy of GQF. In conclusion, dietary GQF and its microbe‐releasable FA represent a natural nutritional strategy, providing a promising preventive and adjuvant approach for metabolic‐related cognitive dysfunction.

## Graphical abstract

Liberation of ferulic acid by gut microbial feruloyl esterase mediates cognitive enhancement of germinated quinoa (GQF) on obese mice. Ferulic acid enriched in GQF is released by gut microbes *Roseburia hominis* and *R. intestinalis* and subsequently mediates the neuroprotective effects of GQF on obesity‐induced cognitive decline by activating hippocampal mitophagy and maintaining synaptic plasticity.

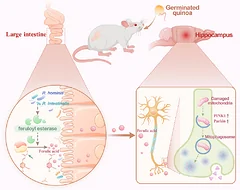

## 1 Introduction

Obesity represents a major global health burden, currently affecting more than one billion individuals worldwide, with its prevalence continuing to rise [1]. Obesity has been identified as a modifiable risk factor for cognitive impairment; conversely, cognitive dysfunction itself can predispose individuals to overeating and the development of obesity [2, 3]. Clinical studies have demonstrated that for every 0.27 kg increase in visceral or 4.52 kg/m^2^ general adiposity, cognitive aging accelerates by approximately 0.7 years [4]. In contrast, a randomized controlled trial reported that for each 1% reduction in body weight or body mass index (BMI) among individuals with obesity, the rate of brain functional decline was reduced by approximately 8.9 months [5]. Among the core cognitive domains, learning and memory are often the earliest and most prominently affected in obese individuals [3, 6]. Accumulating evidence from both animal models and human studies further indicates that excessive consumption of a high‐fat diet (HFD) induces widespread impairments in complex behaviors, with particularly pronounced deficits in learning‐ and memory‐related cognitive functions [7].

The gut‐brain axis facilitates bidirectional communication between the gut microbiota, microbiota‐derived bioactive mediators, and the brain, and plays a critical role in metabolic‐associated cognitive impairment [8]. Through this axis, gut microorganisms directly or indirectly mediate the effects of dietary components on brain function. For example, the cognition‐enhancing effects of rice bran peptides in aged mice can be directly transferred via the gut microbiota, particularly Akkermansia muciniphila [9]. Meanwhile, the protective effects of inulin against offspring learning and memory deficits induced by maternal obesity are indirectly mediated by microbial fermentation products, such as short‐chain fatty acids (SCFAs) [10]. Beyond bacterial fermentation‐derived metabolites, bioactive compounds liberated from the diet by microbial enzymes also play a crucial role in mediating nutritional responses. Zeng et al. demonstrated that the hepatoprotective effects of soybean‐based foods are associated with the microbial release of isoflavone aglycones via β‐glucosidase or β‐galactosidase activity [11]. In the distal gastrointestinal tract, gut microbes employ a diverse enzymatic repertoire to modify poorly digestible dietary components, generating bioactive molecules that mediate interactions with host cells [12]. These sequestered natural products released by bacterial enzymes have been termed “celobiotics” [13]. Identifying the specific microorganisms and enzymatic pathways responsible for celobiotic liberation, and elucidating how these compounds mediate diet‐microbiota‐host and/or diet‐host interactions, will advance our understanding of the mechanisms underlying dietary regulation of host biology and further support the development of personalized nutrition and therapeutic strategies.

Whole grains are widely recognized for their ability to reduce the risk of obesity and obesity‐associated cognitive impairment [14, 15]. Among them, quinoa possesses distinct advantages in the prevention of chronic metabolic disorders due to its rich profile of bioactive nutrients and its status as a nutritionally complete food [16, 17, 18]. In recent years, germination has been shown to markedly enhance the levels of polyphenols and other bioactive compounds in quinoa, thereby further potentiating its health‐promoting properties [19, 20]. An increasing body of evidence indicates that consumption of germinated quinoa (GQF) is closely associated with enhanced antioxidant defense, improved glycemic control, and attenuation of hyperlipidemia [21]. Notably, an intriguing study by Eve et al. demonstrated that germinated brown rice alleviates cerebral ischemia‐induced learning and memory deficits by suppressing neuronal cell injury and activating γ‐aminobutyric acid type A (GABA_A_) receptors, highlighting the substantial potential of germinated grains in cognitive protection [22]. Nevertheless, the efficacy of GQF in ameliorating HFD‐induced obesity and the associated cognitive deficits, as well as the underlying molecular mechanisms, remains to be systematically elucidated.

In this study, we aimed to elucidate the protective mechanisms of GQF intake against obesity‐associated cognitive deficits and to determine the potential involvement of the gut‐brain axis. We found that GQF markedly ameliorated HFD‐induced obesity and cognitive dysfunction, with effects superior to those of non‐germinated quinoa (QF). Mechanistically, dietary GQF intake enriched the Firmicutes bacteria *Roseburia* (including *R. hominis* and *R. intestinalis*), which depolymerized bound ferulic acid (FA) in GQF via feruloyl esterase (FAE) activity. The resulting increase in free FA subsequently contributed to the GQF‐induced enhancement of synaptic function in obese mice by activating hippocampal mitophagy and modulating mitochondrial function, an effect accompanied by the upregulation of the PINK1/Parkin signaling pathway. These findings highlight the importance of diet‐microbiota‐host interactions in dietary nutritional interventions and expand our understanding of Firmicutes‐mediated transformation of dietary polyphenols. Collectively, our results suggest that the microbial release of sequestered natural products (e.g., FA) contributes to the beneficial effects of whole‐grain intake on obesity and metabolic‐associated cognitive impairment.

## 2 Results

### 2.1 GQF Supplementation Ameliorates Learning and Memory Deficits and Restores Hippocampal Synaptic Plasticity in HFD‐Induced Obese Mice

To investigate the effects of GQF on obesity‐associated cognitive impairment, mice were fed an HFD supplemented with different doses of GQF or QF (Figure 1A). Compared with the HFD group, low‐dose GQF (LGQF), high‐dose GQF (HGQF), and high‐dose QF (HQF) interventions significantly attenuated body weight gain and adipose tissue accumulation without altering caloric energy intake, with the HGQF group exhibiting a phenotype most comparable to that of control mice (Figure S1A–I). A battery of behavioral tests was conducted to assess hippocampus‐dependent learning and memory as well as activities of daily living. In the open‐field test, obese mice displayed a marked reduction in total locomotor distance and a decreased tendency to explore the center area, indicating impaired spontaneous activity (Figure 1B,C). Dietary intervention with HGQF significantly restored locomotor activity in obese mice, whereas HQF and LGQF exerted only partial amelioration, yielding significant increases in total distance but a non‐significant trend in center distance (Figure 1B,C). Consistently, mice receiving HGQF exhibited increased spontaneous alternation behavior in the Y‐maze test and higher discrimination indices in the novel object recognition test, reflecting improvements in working memory and short‐term memory (Figure 1D–G,J). The Barnes maze was subsequently employed to evaluate spatial learning and memory. HGQF supplementation significantly reduced escape latency during the experiment, and on the probe trial day, HGQF‐treated mice showed a markedly increased number of visits to the target quadrant (Figure 1H,I,K,L). Collectively, these results demonstrate that dietary GQF effectively ameliorates obesity‐induced cognitive deficits, with HGQF exerting the most pronounced effects, followed by LGQF and HQF.

**FIGURE 1:**
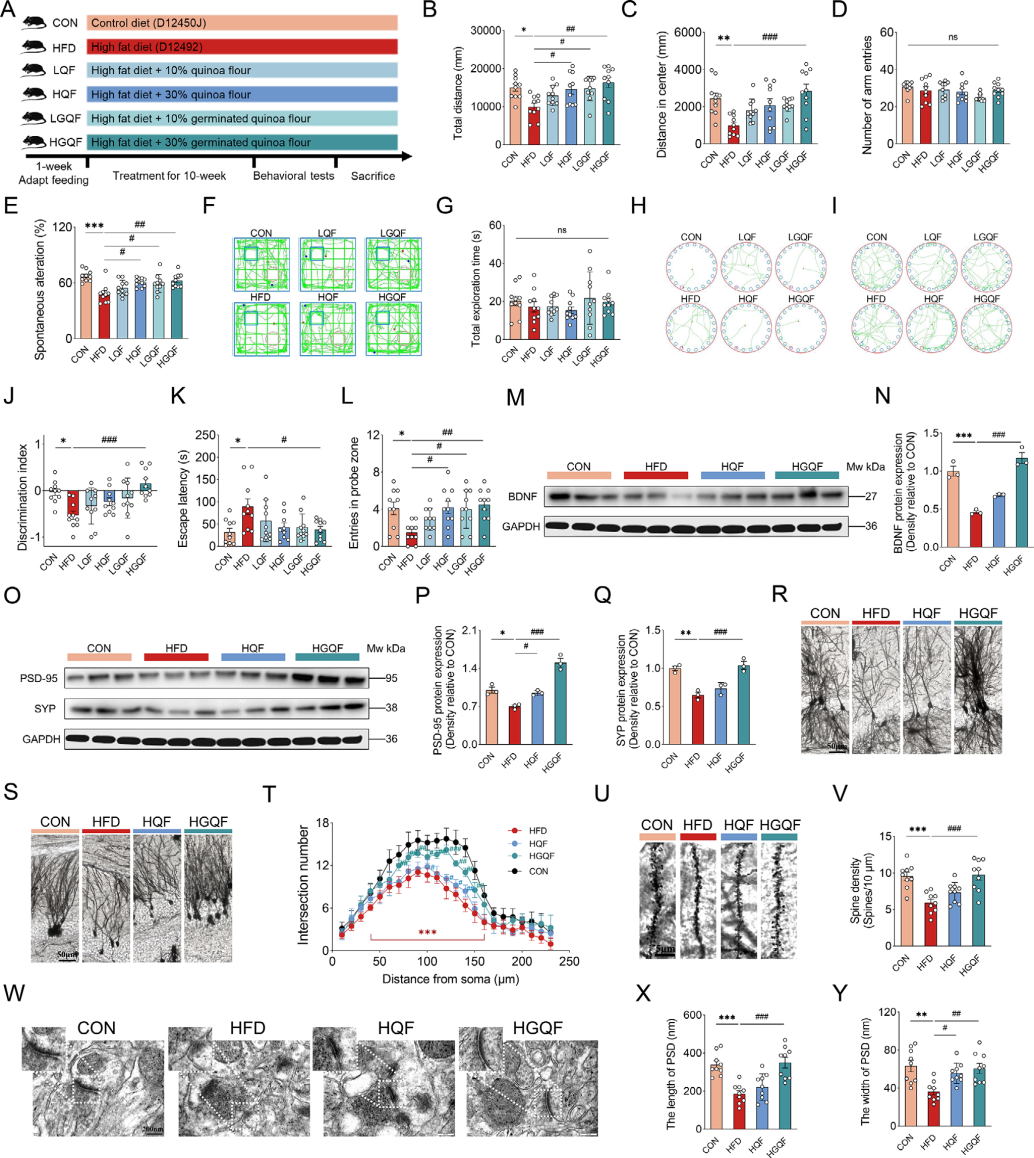
GQF ameliorates learning and memory deficits and restores hippocampal synaptic plasticity in HFD‐induced obese mice. (A) Schematic diagram of the experimental procedures. (B,C) Total distance (B) and distance in center (C) in the open field test (*n* = 10). (D,E) Total number of arm entries (D) and spontaneous alternation (E) in Y‐maze test (*n* = 10). (F,G,J) The representative track images (F), total exploration time (G), and discrimination index (J) of the novel object recognition test (*n* = 10). (H,I, K,L) The representative track images (H,I), escape latency (K), and entries in probe zone (L) of Barnes maze during training and testing (*n* = 10). (M−Q) Protein expression levels of hippocampal BDNF, PSD‐95, and SYP in mice (*n* = 3). Western Blots in Figure M and O were derived from the same batch of mouse tissue samples from identical mouse groups, yielding identical GAPDH loading control bands. (R,S) Representative images of Golgi staining of hippocampal pyramidal cells (R) and granule cells (S). Scale bar, 50 µm. (T) Complexity analysis of pyramidal neuron dendrites (*n* = 9 slices from 3 mice). (U) Representative images of dendritic spines of hippocampal neurons. Scale bar, 5 µm. (V) Dendrite spine density of hippocampal neurons (*n* = 9 slices from 3 mice). (W) Representative images of synaptic ultrastructure by transmission electron microscope. Scale bar, 200 nm. (X,Y) The length (X) and the width (Y) of PSD (*n* = 9 slices from 3 mice). Data are presented as the mean ± SEM. ^*^ *p* < 0.05, ^**^ *p* < 0.01, and ^***^ *p* < 0.001 vs. CON group. ^#^ *p* < 0.05, ^##^ *p* < 0.01, and ^###^ *p* < 0.001 vs. HFD group.

To elucidate the cellular and molecular basis underlying the cognitive improvements conferred by GQF, neuronal function and synaptic plasticity were examined in the hippocampus, a brain region critical for cognition and memory. Hematoxylin and eosin (H&E) staining revealed that HGQF intervention restored hippocampal neuronal morphology to near‐normal levels in obese mice, whereas LGQF and HQF only partially alleviated neuronal abnormalities in the CA1 and CA3 regions (Figure S1J). Moreover, HGQF supplementation significantly upregulated hippocampal mRNA expression of neurotrophic factors involved in neuronal growth (including *Bdnf*, *Ngf*, *Nt3*, and *Nt4*) as well as synaptic plasticity‐related markers (*Psd* *‐* *95* and *Syp*) (Figure S1K–N,Q,R). Based on these findings, four groups (CON, HFD, HQF, and HGQF) were selected for further mechanistic analyses. Compared with HFD‐fed mice, HGQF markedly increased hippocampal protein levels of BDNF, PSD‐95, and SYP (Figure 1M–Q and Figure S1O,P). Golgi staining revealed that chronic HFD consumption severely impaired dendritic arborization in the hippocampus, whereas HGQF more effectively enhanced dendritic complexity in obese mice compared with HQF, as verified by Sholl analysis of pyramidal neurons (Figure 1R–T). Dendritic spine density was further quantified from Golgi‐stained images, and HGQF intervention significantly increased spine density (Figure 1U,V). Ultrastructural analysis of hippocampal synapses showed that HGQF supplementation increased both the average length and width of the postsynaptic density (PSD) (Figure 1W–Y). Taken together, these findings provide compelling evidence that dietary GQF robustly ameliorates obesity‐associated cognitive impairment and synaptic plasticity deficits, with effects superior to those observed with QF.

### 2.2 GQF Supplementation Reshapes Gut Microbiota and Selectively Enriches *Roseburia*‐Related Beneficial Genera in HFD‐Induced Obese Mice

Given that the gut microbiota is highly dependent on dietary substrates, we next investigated the effects of GQF supplementation on microbial community characteristics in obese mice. Analysis of 16S rRNA gene‐based α‐diversity revealed that both GQF and QF dietary interventions significantly increased bacterial richness and diversity in HFD‐fed mice (Figure 2A–E). Notably, microbial species richness and diversity in the HGQF group exceeded those observed in both the CON and HQF groups (Figure 2A–E), highlighting the superior efficacy of GQF in restoring gut microbial composition in obese mice. Principal component analysis (PCA) demonstrated a clear separation of fecal microbial communities among the four experimental groups (Figure 2F). Compared with the HFD group, HGQF supplementation induced marked alterations in overall gut microbiota composition (Figure 2H and Figure S2A–C). In particular, HGQF intervention restored the Firmicutes‐to‐Bacteroidetes ratio in obese mice (Figure 2H,I). Linear discriminative analysis (LDA) effect size (LEfSe) analysis of the taxonomic alternations further revealed that *Clostridia*, *Sporobacter*, *Ruminococcus*, *Butyricicoccus*, *Odoribacter*, and *Roseburia* were the characteristic differential genera in the HGQF intervention group. The discriminant feature genera identified in the HQF group include *Coprococcus*, *Parvibacter*, *Intestinimonas*, and *Acetivibrio* (Figure 2G and Figure S2D). Importantly, compared with the HFD and HQF groups, HGQF selectively enriched the abundance of *Roseburia* *, Butyricicoccus*, *Ruminococcus*, and *Odoribacter*, genera that were not significantly represented in the CON group (Figure 2J–N and Figure S2E–G). Collectively, these observations indicate that dietary GQF supplementation effectively reshapes gut microbial community structure in obese mice, which may contribute to the neuroprotective effects associated with GQF intervention.

**FIGURE 2:**
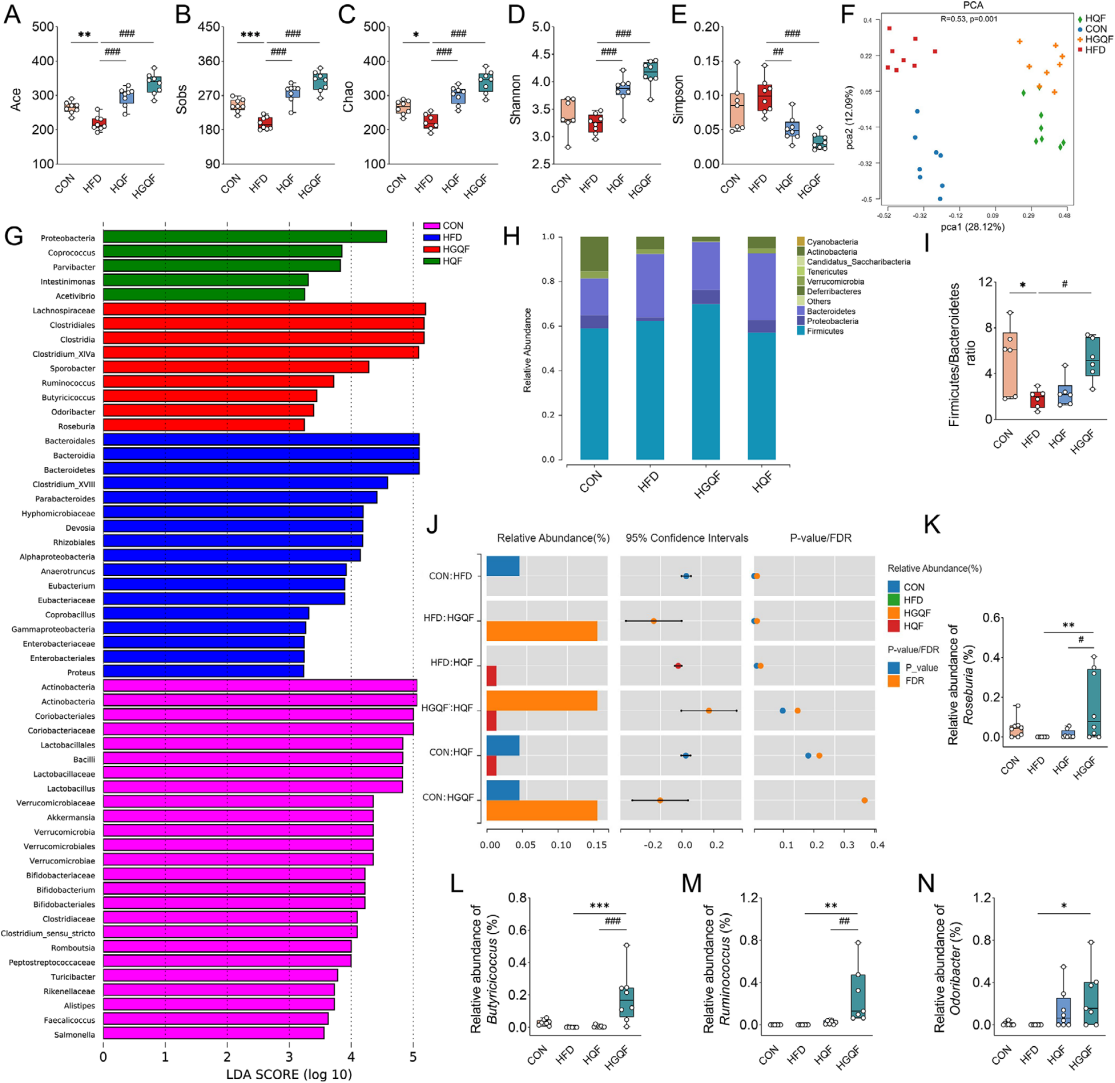
GQF alters the gut microbiota composition in HFD‐induced obese mice. (A) Ace index. (B) Sobs index. (C) Chao index. (D) Shannon index. (E) Simpson index. (F) Plot of principal components analysis. (G) Linear discriminant analysis (LDA) of microbial composition based on differentially expressed taxa (LDA scores > 2.0 and adjusted *p* < 0.05). (H) Averaged relative abundance of bacteria at the phylum level. (I) The ratio of Firmicutes / Bacteroidetes. (J) Pairwise comparisons of gut microbial taxa abundance between experimental groups of *Roseburia* at the genus level. Non‐parametric comparisons across four groups were performed using the Kruskal‐Wallis H test. For taxa with a significant overall effect (*p* < 0.05), post‐hoc pairwise comparisons were conducted using Dunn's test with the Benjamini‐Hochberg FDR correction to control for multiple comparisons. (K−N) The relative abundance of *Roseburia* (K), *Butyricicoccus* (L), *Ruminococcus* (M), and *Odoribacter* (N) in mouse feces. Data are presented as the median ± interquartile range. *n* = 8 mice per group. (A−E) and (I) ^*^ *p* < 0.05, ^**^ *p* < 0.01, and ^***^ *p* < 0.001 vs. CON group. ^#^ *p* < 0.05, ^##^ *p* < 0.01, and ^###^ *p* < 0.001 vs. HFD group. (K−N) ^*^ *p* < 0.05, ^**^ *p* < 0.01, and ^***^ *p* < 0.001 vs. HFD group. ^#^ *p* < 0.05, ^##^ *p* < 0.01, and ^###^ *p* < 0.001 vs. HQF group.

### 2.3 The Neuroprotective Effects of GQF Depend on the Microbiota‐Substrate Synergy

Given the pronounced modulatory effects of GQF on gut microbial composition and diversity, we next sought to determine whether the gut microbiota directly mediates the GQF‐induced improvements in learning and memory deficits in obese mice, thereby elucidating potential mechanisms underlying its neuroprotective effects. To this end, fecal microbiota transplantation (FMT) experiments were performed (Figure 3A). Following gut microbiota depletion and reconstruction, recipient mice were fed an HFD either containing 30% GQF and receiving fecal microbiota from HGQF donor mice (FGQ group) or lacking GQF but receiving the same donor microbiota (FGH group). FCON and FHFD groups served as the control and model groups, respectively. High‐throughput 16S rRNA gene sequencing of fecal samples from recipient mice revealed that both FGH and FGQ interventions increased microbial species richness and diversity in obese mice, with a more pronounced effect observed in the FGQ group (Figure 3B–G). β‐Diversity analysis based on Bray–Curtis distances demonstrated distinct gut microbial community structures among the different intervention groups (Figure 3H). Notably, characteristic genera enriched in HGQF donor mice, including *Roseburia* *, Clostridia*, *Ruminococcus*, and *Odoribacter*, were also significantly enriched in the gut microbiota of both FGH‐ and FGQ‐treated recipient mice, with *Roseburia* showing particularly robust enrichment in both groups (Figure 3I and Figure S3A,B). These results reveal the successful reshaping of the gut microbiota in recipient mice via FMT.

**FIGURE 3:**
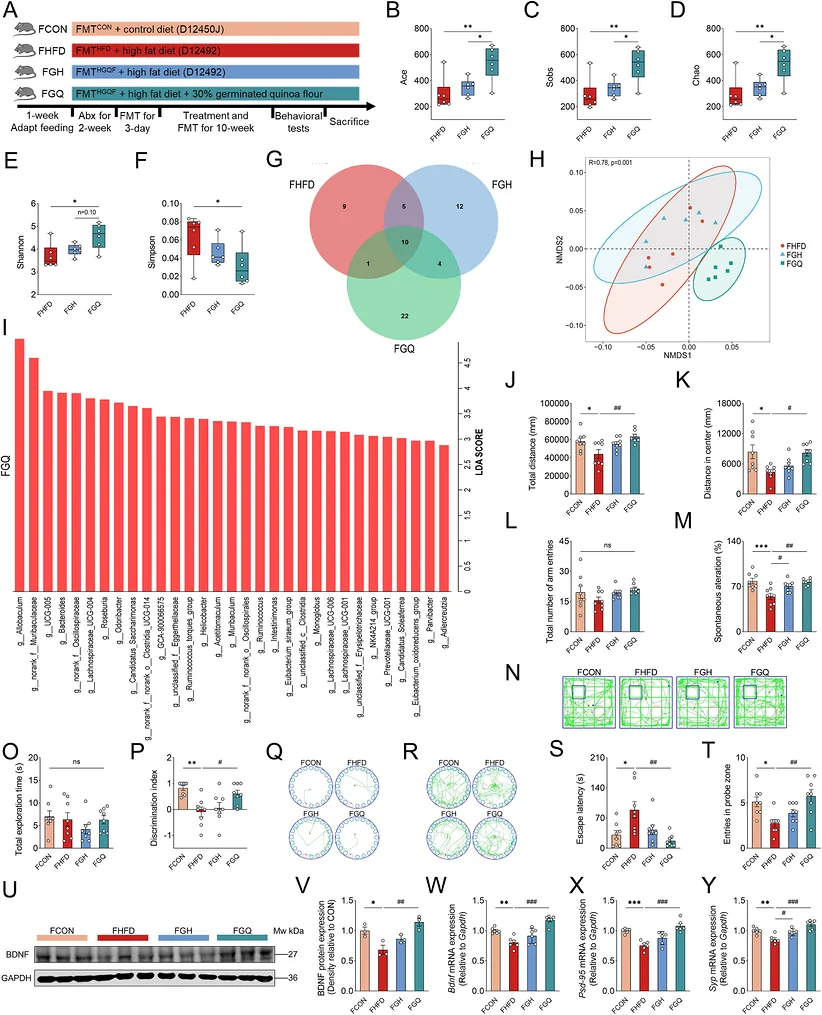
Effect of fecal microbiota transplantation on gut microbiota and cognitive impairment in HFD‐induced obese mice. (A) Schematic diagram of the experimental procedures. (B) Ace index (*n* = 6). (C) Sobs index (*n* = 6). (D) Chao index (*n* = 6). (E) Shannon index (*n* = 6). (F) Simpson index (*n* = 6). (G) OUT Venn diagram. (H) Non‐metric multidimensional scaling analysis (*n* = 6). (I) Linear discriminant analysis (LDA) of microbial composition based on differentially expressed taxa (LDA scores > 2.0 and adjusted *p* < 0.05) (*n* = 6). (J,K) Total distance (J) and distance in the center (K) in the open field test (*n* = 8). (L,M) Total number of arm entries (L) and spontaneous alternation (M) in Y‐maze test (*n* = 8). (N−P) The representative track images (N), total exploration time (O), and discrimination index (P) of novel object recognition test (n = 8). (Q−T) The representative track images (Q,R), escape latency (S), and entries in probe zone (T) of the Barnes maze during training and testing (*n* = 8). (U,V) Protein expression levels of hippocampal BDNF in mice (*n* = 3). (W−Y) Relative levels of *Bdnf*, *Psd‐95*, and *Syp* mRNA in hippocampus (n = 6). (B−F) Data are presented as the median ± interquartile range. ^*^ *p* < 0.05, ^**^ *p* < 0.01. (J−Y) Data are presented as the mean ± SEM. ^*^ *p* < 0.05, ^**^ *p* < 0.01, and ^***^ *p* < 0.001 vs. FCON group. ^#^ *p* < 0.05, ^##^ *p* < 0.01, and ^###^ *p* < 0.001 vs. FHFD group.

In terms of obesity‐related phenotypes, body weight changes and adipose tissue accumulation in FGQ‐treated mice were comparable to those in the control group, whereas FGH intervention only partially alleviated the obese phenotype (Figure S3C–H). Across a battery of behavioral tests, we observed that only FGQ‐treated recipient mice exhibited behavioral patterns largely resembling those of HGQF donor mice (Figure 3J–T). Histopathological analysis revealed that, compared with the FCON group, FHFD mice displayed reduced neuronal density, nuclear condensation, and abnormal neuronal morphology in the hippocampus, particularly with loosely organized neuronal architecture in the CA regions; these pathological changes were markedly reversed by FGQ intervention (Figure S3I). Consistently, relative to FHFD mice, expression levels of the neurotrophic factor BDNF and the synaptic proteins PSD‐95 and SYP were not significantly altered in the FGH group but were significantly upregulated following FGQ treatment (Figure 3U–V and Figure S3M–O). These findings were further supported by increased hippocampal mRNA expression of neurotrophic factors (*Bdnf*, *Ngf*, *Nt3*, and *Nt4*) and synaptic markers (*Psd‐95* and *Syp*) (Figure 3W–Y and Figure S3J–L). Collectively, these results indicate that gut microbiota derived from HGQF‐treated mice synergizes with dietary GQF to effectively ameliorate obesity‐associated cognitive impairment and hippocampal synaptic dysfunction. Collectively, these results indicate that FMT with HGQF‐derived flora alone (FGH group) failed to fully recapitulate the neuroprotective effects of GQF. Notably, mice in the FGH group were not supplemented with GQF‐rich bound FA substrates, so the transplanted gut microbiota lacked sufficient precursors to produce and release free FA. By contrast, the combined intervention of FMT and GQF dietary supplementation achieved the best therapeutic efficacy, demonstrating that the neuroprotective effects of GQF rely on the synergistic action of gut microbiota and dietary functional substrates.

### 2.4 FA, Enriched in GQF, is a Key Mediator of GQF‐Induced Protection Against Obesity‐Associated Cognitive Impairment

To comprehensively characterize the chemical composition of GQF and identify potential bioactive mediators (with a focus on metabolites enriched in GQF relative to QF) responsible for its differential neuroprotective effects, we performed metabolomic profiling of GQF and QF samples (Figure S4A). Substantial differences in chemical composition were observed between GQF and QF (Figure S4B–D). Orthogonal partial least squares‐discriminant analysis (OPLS‐DA) identified a total of 198 metabolites with significantly different abundances between the two samples, of which 189 metabolites were upregulated in GQF (Figure 4A and Figure S4E). Among these upregulated metabolites, phenolic compounds constituted the largest category (46 metabolites), followed by amino acids and their derivatives (31 metabolites). KEGG pathway enrichment analysis revealed that the differential metabolites were involved in 54 metabolic pathways, with phenylalanine metabolism showing the most significant enrichment (Figure 4B). Notably, phenylalanine metabolism is a central pathway for the biosynthesis of phenolic compounds, particularly through the downstream shikimate pathway, which serves as the primary route for the production of phenolic acids such as FA, *p*‐coumaric acid, and chlorogenic acid. Heatmap analysis further demonstrated that the phenolic compounds significantly enriched in GQF were predominantly phenolic acids and flavonoids (Figure 4C). Consistent with this observation, fold‐change analysis identified the top 24 markedly changed metabolites, including 8 polyphenols, 4 nucleotides and derivatives, 2 amino acids and derivatives, 2 organic acids and derivatives, 2 alkaloids, 3 sugars and derivatives, and 3 other metabolites, demonstrating that phenolic compounds dominated the intergroup metabolic differences (Figure 4D). Of these polyphenolic metabolites, the majority were significantly elevated. Notably, FA exhibited the highest relative content at 0.27% (Table S1). Collectively, these results indicate that germination primarily enhances phenylalanine metabolism in quinoa, leading to a marked enrichment of FA and other phenolic compounds, which may provide a critical material basis for the neuroprotective effects of GQF.

**FIGURE 4:**
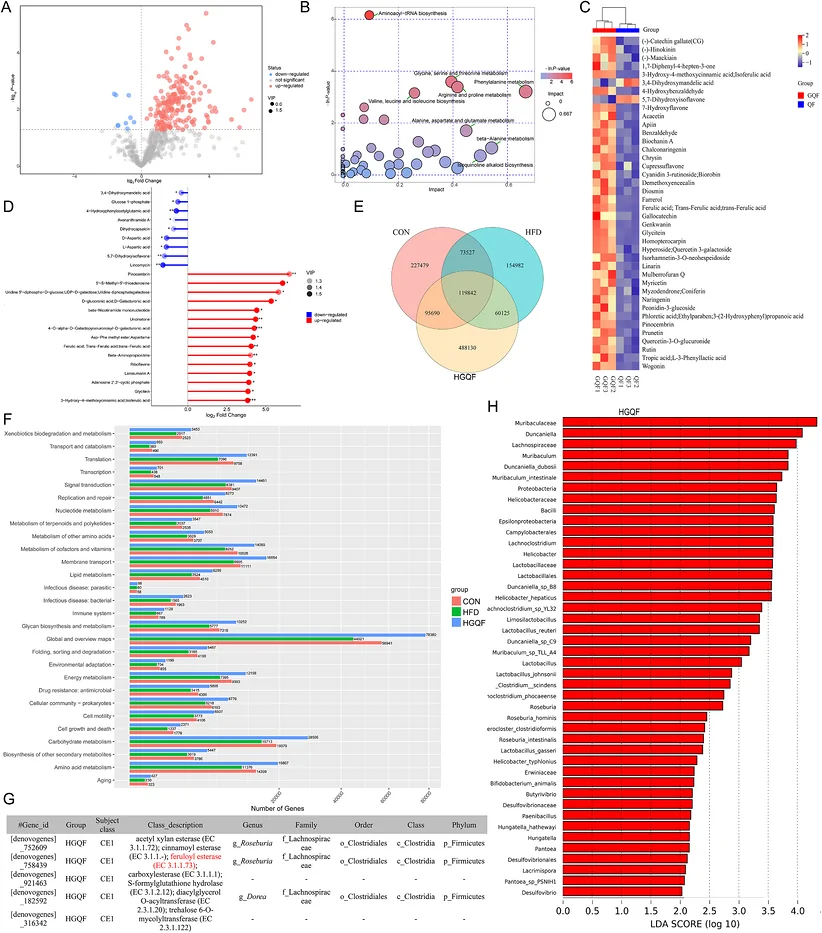
Ferulic acid (FA) is a key mediator underlying the protective effects of GQF against cognitive impairment in obese mice. (A) Volcano plot displaying 198 differentially expressed metabolites for GQF vs. QF (*n* = 3). (B) KEGG pathway analysis of differential biological processes for GQF vs QF. (C) Heatmap of hierarchical clustering analysis for phenolic compounds in differential metabolites (*n* = 3). (D) Matchstick analysis for key differential metabolites of GQF vs QF. (E) Fecal metagenome Venn diagram in mice (CON, HFD, HGQF groups) (*n* = 5). (F) Functional annotation analysis of fecal metagenome based on the KEGG database. (G) Functional annotation and genomic characteristic analysis of gut microbiota based on fecal metagenome. (H) Linear discriminant analysis (LDA) of microbial composition based on differentially expressed taxa (LDA scores > 2.0 and adjusted *p* < 0.05).

Based on the above results and prior literature [20, 21], we further performed targeted quantitative analysis of 34 major polyphenolic compounds in GQF and QF (Table S2). The predominant polyphenols in QF were rutin, vanillic acid, 2‐hydroxycinnamic acid, kaempferol‐3‐O‐rutinoside, FA, trans‐4‐hydroxycinamic acid, isoquercitrin, and benzoic acid. For GQF, the main enriched polyphenols were FA, trans‐4‐hydroxycinamic acid, kaempferol‐3‐O‐rutinoside, rutin, caffeic acid, vanillic acid, isoferulic acid, and 4‐hydroxybenzoic acid. Notably, FA was the most abundant polyphenol in GQF (1299.36 µg/g), occurring predominantly in a bound form, and its content was increased by 2.29‐fold compared with QF. FA has been extensively studied for its protective effects on human health, with documented benefits in obesity, cardiovascular and cerebrovascular diseases, Alzheimer's disease, and cancer [23]. Based on these findings, we hypothesized that FA represents a key bioactive mediator underlying the cognitive benefits of GQF. However, FA in cereal grains is primarily covalently bound to cell wall polysaccharides (such as cellulose, lignin, and hemicellulose) via ester linkages and typically requires the action of specific gut microbial enzymes to be released and subsequently absorbed [24, 25]. Therefore, elucidating the microbial mechanisms responsible for the colonic release of FA and identifying the microorganisms involved may be critical for understanding the neuroprotective mechanisms of GQF.

### 2.5 GQF‐Induced *Roseburia* Enrichment Promotes FA Release from GQF via Bacterial FAE

To investigate the microbial mechanisms underlying the colonic release of FA from GQF, metagenomic sequencing was performed to characterize the gut bacterial genomic profiles of mice in the CON, HFD, and HGQF groups. Significant differences in bacterial gene composition were observed among the experimental groups, with GQF supplementation markedly increasing gut bacterial genomic diversity (Figure 4E and Figure S4F–H). KEGG annotation revealed that genes involved in carbohydrate metabolism were the most abundant across all samples, with particularly higher enrichment in the HGQF group compared with the HFD and CON groups (Figure S4I and Figure 4F). These findings suggest that GQF intervention enhances the capacity of the gut microbiota for substrate degradation and carbohydrate metabolism. The release of complex FA from cereal matrices requires the coordinated action of multiple carbohydrate‐active enzymes (CAZymes) [26]. FAE (E.C. 3.1.1.73), a subfamily of carbohydrate esterases (CEs), is known to specifically hydrolyze ester bonds between FA and plant cell wall polysaccharides [27]. Accordingly, functional annotation based on the CAZy database revealed that the gut bacterial genomes of GQF‐treated mice were enriched in genes encoding CAZymes, including glycoside hydrolases (GHs), glycosyltransferases (GTs), and CEs (Figure S4J). Notably, FAE‐encoding genes exhibited pronounced clustering in the bacterial genomes of GQF‐supplemented mice (Figure 4G). Further taxonomic annotation identified FAE gene sequences within two gut bacterial genera, *Roseburia* and *Dorea* (Figure 4G). LDA demonstrated that *Roseburia*, including *R. hominis* and *R. intestinalis*, was specifically enriched in the gut microbiota of GQF‐treated mice (Figure 4H and Figure S4K,L), consistent with the 16S rRNA gene sequencing results. Collectively, these findings suggest that GQF supplementation promotes the enrichment of *R. hominis* and *R. intestinalis*, which may drive FAE expression and further facilitate the microbial release of bound FA from GQF.

In vitro assays further demonstrated that *R. hominis* and *R. intestinalis* are capable of producing functional FAE (Figure 5A–C). Whole‐genome sequence analysis confirmed that the genomes of both *R. hominis* and *R. intestinalis* harbor open reading frames (ORFs) encoding FAE (Figure 5D,E). In vivo, dietary GQF supplementation significantly increased fecal FAE activity (Figure 5F). Consistently, RT‐qPCR analysis revealed a marked upregulation of *Fae* mRNA expression following GQF intervention (Figure S5A). The functional role of *Roseburia* in FA release was further validated using in vitro anaerobic fermentation assays. Briefly, 10^6^ CFU of each bacterial strain were incubated anaerobically with 10 mL of GQF suspension (5 mg/mL) for 72 h. Targeted quantification of free FA in the culture supernatants was performed using a Triple Quad 5500+ QTRAP system. Compared with control conditions, substantial amounts of free FA were detected in GQF suspensions fermented with either *R. hominis* or *R. intestinalis* (Figure 5G–I). We also obtained evidence indicating that *Roseburia* strains do not further utilize or degrade FA under the tested conditions. Supplementation of FA during the cultivation of *R. hominis* or *R. intestinalis* did not promote bacterial growth or enhance FA production. Moreover, when FA was provided as the sole energy source, neither strain exhibited detectable cell proliferation, nor were significant changes in FA levels observed (Figure S5B–D). Changes in protein concentrations in the culture media further supported these findings (Figure S5E). Targeted mass‐spectrometry analysis revealed elevated FA levels in multiple biological compartments, including the cecum, liver, brain, serum, feces, and cecal contents, of mice fed a GQF‐supplemented diet (Figure 5J–O). Importantly, the relative abundances of *R. hominis* and *R. intestinalis* showed a positive correlation with brain FA concentrations in GQF‐intervened obese mice (Figure 5P,Q). Together, these results indicate that dietary GQF intake promotes microbial FA release by enriching *Roseburia*, thereby increasing FA availability and distribution in the brain and other peripheral tissues.

**FIGURE 5:**
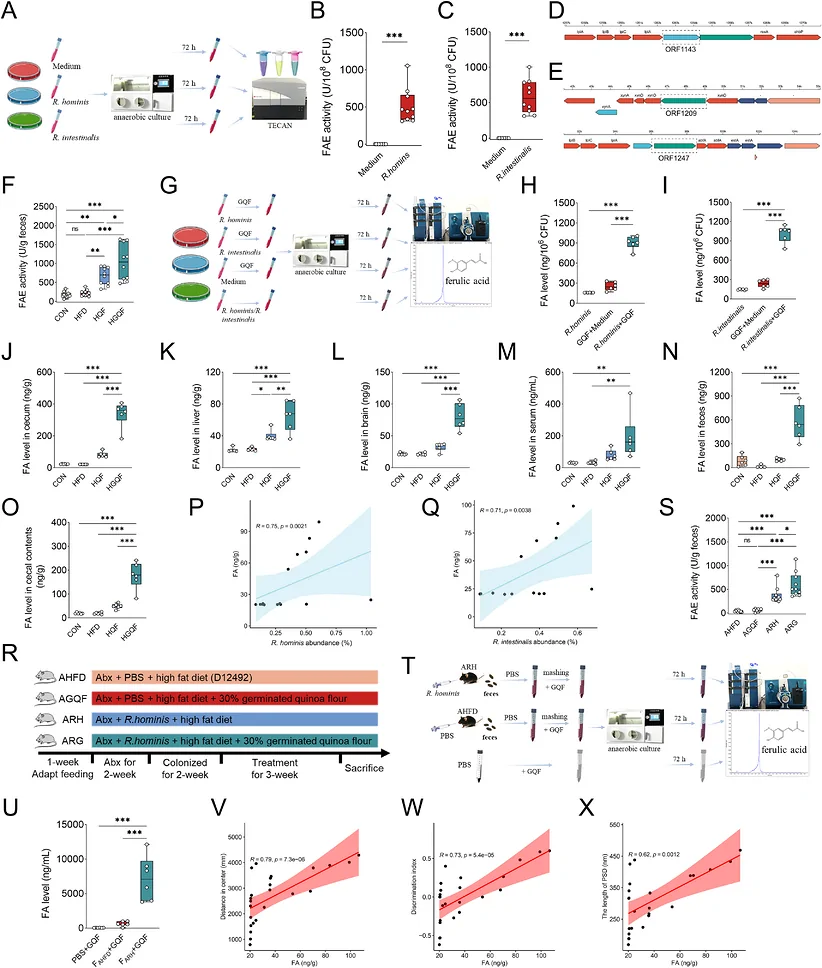
GQF‐induced *Roseburia* enrichment promotes FA release from GQF via bacterial feruloyl esterase (FAE). (A−C) Assay for FAE activity in *R. hominis* and *R. intestinalis* (*n* = 10). (D) The genome of *R. hominis* contained ORF1143 encoding FAE large subunit. (E) The genome of *R. intestinalis* contained ORF1209 and ORF1247 encoding FAE large subunit. The length and direction of arrows on the map represent the length and coding direction of the genes respectively. (F) Determination of FAE activity in mice feces (CON, HFD, HQF, HGQF groups) (*n* = 10). (G−I) The levels of FA in GQF cultural supernatant were quantified using UPLC‐MS/MS. GQF was resuspended in medium (5 mg/mL), followed by incubation with *R. hominis* (1 × 10^6^ CFU/mL) or *R. intestinalis* (1 × 10^6^ CFU/mL) for 72 h under anaerobic conditions (n = 6). (J−O) The levels of FA were quantified in mice cecum (J), liver (K), brain (L), serum (M), feces (N), and cecal contents (O) using UPLC‐MS/MS (*n* = 6). (P,Q) Correlation analysis of the abundance of *R. hominis* (P) and *R. intestinalis* (Q) with the FA levels in mice brain. (R) Schematic diagram of the experimental procedures. (S) Determination of FAE activity in mice feces (AHFD, AGQF, ARH, ARG groups) (*n* = 10). (T−U) The levels of FA were quantified in GQF after fermentation by mouse fecal bacteria using UPLC‐MS/MS. Mouse feces were mashed and resuspended in PBS (0.125 g/mL), followed by incubation with GQF (50 mg) for 72 h under anaerobic conditions (*n* = 6). (V−X) Correlation analysis of distance in center of the open field test (V), discrimination index of novel object recognition test (W), and the length of PSD (X) with the FA levels in mice brain. Data are presented as the median ± interquartile range. ^*^ *p* < 0.05, ^**^ *p* < 0.01, and ^***^ *p* < 0.001.

Based on the above findings, we conducted in vivo experiments to evaluate the ability of *Roseburia* to mediate FA release from GQF. Given its higher LDA score, *R. hominis* was selected for in vivo validation. Four groups of C57BL/6 mice were first treated with a broad‐spectrum antibiotic cocktail in drinking water to deplete the endogenous gut microbiota. Two groups were subsequently colonized with *R. hominis*, after which mice were allowed ad libitum access to an HFD containing either 30% GQF or no GQF for 21 days. The remaining two groups were maintained in a pseudo‐germ‐free state and gavaged with PBS, while being fed the same HFDs with or without 30% GQF (Figure 5R). RT‐qPCR analysis of fecal samples confirmed successful colonization of *R. hominis* in recipient mice (Figure S5F). Colonization with *R. hominis* markedly enhanced in vivo expression of FAE (Figure 5S and Figure S5G). Ex vivo fecal fermentation assays further demonstrated that incubation of fecal microbiota from *R. hominis*‐colonized mice with sterile GQF significantly increased FA release (Figure 5T–U). Consistently, elevated FA levels were detected exclusively in the cecum, liver, brain, serum, feces, and cecal contents of GQF‐fed mice colonized with *R. hominis*, whereas such increases were not observed in non‐colonized mice (Figure S5H–N). Moreover, in GQF‐treated mice, brain FA concentrations exhibited a positive correlation with behavioral cognitive parameters and synaptic plasticity‐related markers (Figure 5V–X and Figure S5O–R). Collectively, these findings support the hypothesis that the beneficial effects of GQF against obesity‐associated cognitive impairment are, at least in part, dependent on the microbial release of FA.

### 2.6 *Roseburia*‐Driven Eelevated FA Availability Ameliorates Learning and Memory Deficits and Restores Synaptic Plasticity in Obese Mice

To determine whether *Roseburia*‐driven elevated FA availability contributes to GQF's effects on cognitive function and synaptic plasticity, we administered FA directly to HFD‐fed mice via oral gavage at doses of 10 mg/kg/day or 40 mg/kg/day for 10 weeks (Figure 6A). This experimental design was intended to evaluate the effects of FA before and after microbial liberation from its bound form, independent of other components present in GQF. The 10 mg/kg/day dose was selected to approximate the estimated daily intake of free FA derived from consumption of an HFD supplemented with 30% GQF, based on measured free FA content in GQF. The 40 mg/kg/day dose was chosen to approximate the estimated total daily intake of both free and bound FA from the same diet, calculated from quantified levels of free and bound FA in GQF. Control and HFD model mice received saline by gavage. Oral FA administration at the higher dose significantly attenuated HFD‐induced obesity phenotypes without affecting caloric energy intake (Figure S6A–I). Behavioral assessments demonstrated that FA supplementation ameliorated learning and memory deficits in obese mice, with the higher dose producing more pronounced improvements (Figure 6B–L). Compared with the HFD group, high‐dose FA (HFA) markedly reduced neuronal damage and prevented the decline in neurotrophic protein expression (Figure 6M–O and Figure S6J–O). Golgi staining further revealed that HFA increased neuronal dendritic complexity and dendritic spine density (Figure 6P–R and Figure S6P,Q). Consistent with these structural changes, high‐dose FA—but not low‐dose FA—significantly alleviated synaptic plasticity impairments in obese mice, as evidenced by increased length and width of the hippocampal PSD and elevated expression of synaptic proteins (Figure 6S–Y and Figure S6R). These effects closely mirrored those observed following GQF intervention. Collectively, these findings indicate that FA, once released from its bound form by gut microbiota, contributes to the cognitive benefits of GQF. Thus, the neuroprotective effects of GQF against obesity‐associated cognitive impairment are mediated, at least in part, by FA.

**FIGURE 6:**
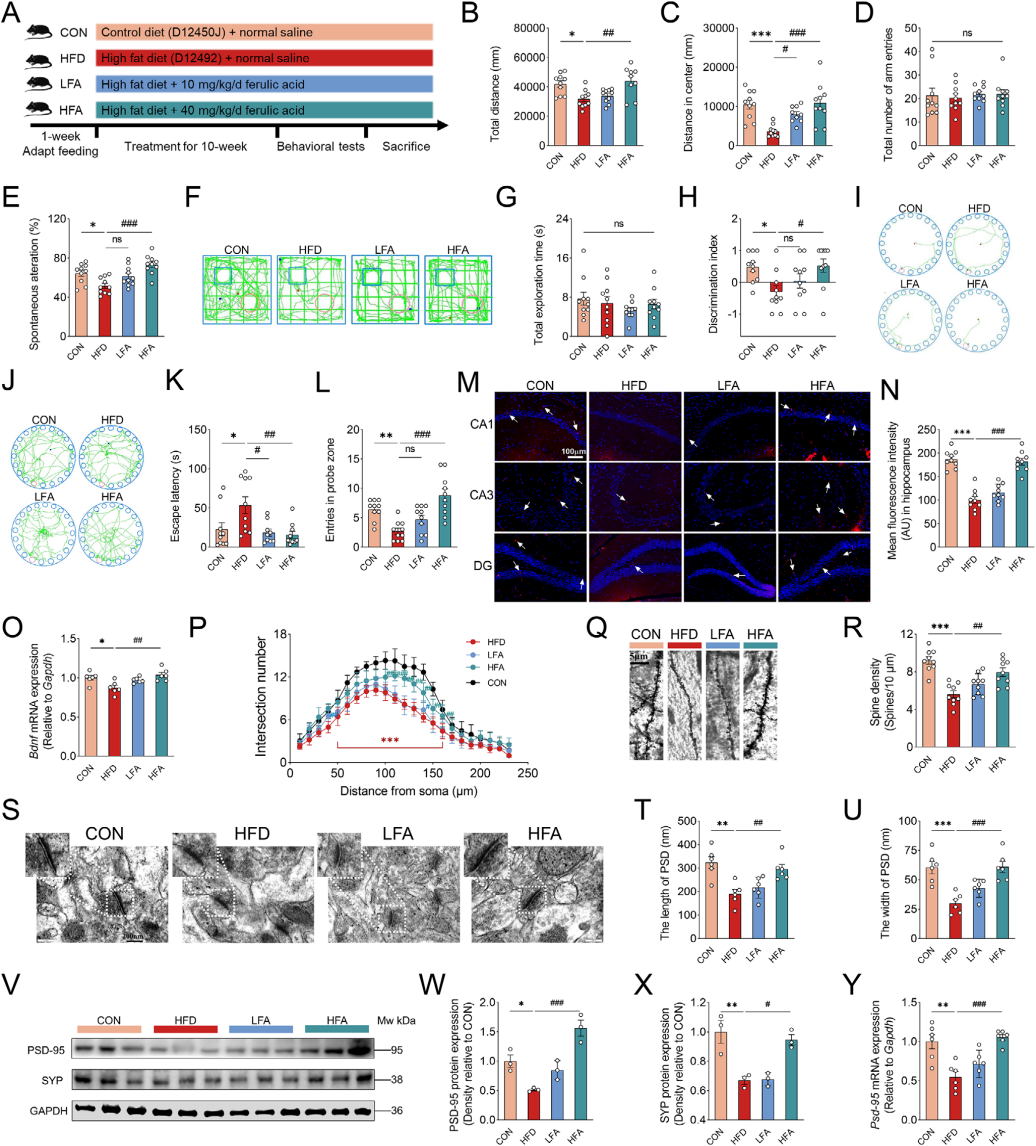
FA ameliorates learning and memory deficits and restores synaptic plasticity in HFD‐induced obese mice. (A) Schematic diagram of the experimental procedures. (B,C) Total distance (B) and distance in center (C) in the open field test (*n* = 10). (D,E) Total number of arm entries (D) and spontaneous alternation (E) in Y‐maze test (*n* = 10). (F–H) The representative track images (F), total exploration time (G), and discrimination index (H) of novel object recognition test (*n* = 10). (I−L) The representative track images (I,J), escape latency (K), and entries in probe zone (L) of the Barnes maze during training and testing (*n* = 10). (M) Representative immunofluorescence staining images of BDNF from hippocampal sections. Scale bar, 100 µm. The arrowheads stand for the representative BDNF. (N) Quantification of the BDNF area based on immunofluorescence staining sections by ImageJ software in the hippocampus (*n* = 9 slices from 3 mice). (O) Relative levels of *Bdnf* mRNA in hippocampus (*n* = 6). (P) Complexity analysis of pyramidal neuron dendrites (*n* = 9 slices from 3 mice). (Q) Representative images of dendritic spines of hippocampal neurons. Scale bar, 5 µm. (R) Dendrite spine density of hippocampal neurons (*n* = 9 slices from 3 mice). (S) Representative images of synaptic ultrastructure by transmission electron microscope. Scale bar, 200 nm. (T−U) The length (T) and the width (U) of PSD (*n* = 6 slices from 3 mice). (V−X) Protein expression levels of hippocampal PSD‐95 and SYP in mice (*n* = 3). (Y) Relative levels of *Psd‐95* mRNA in hippocampus (*n* = 6). Data are presented as the mean ± SEM. ^*^ *p* < 0.05, ^**^ *p* < 0.01, and ^***^ *p* < 0.001 vs. CON group. ^#^ *p* < 0.05, ^##^ *p* < 0.01, and =*p* < 0.001 vs. HFD group.

### 2.7 FA Mediates GQF's Protective Effects on Synaptic Plasticity by Regulating Hippocampal Synaptic Transmission‐Related Genes

To elucidate the molecular mechanisms by which FA mediates GQF's protective effects on synaptic plasticity and cognitive function, we performed comprehensive transcriptomic profiling of the hippocampus. Both HFD and GQF (HGQF group) exerted pronounced effects on hippocampal gene expression (Figure S7A). Differentially expressed gene (DEG) analysis (FDR‐adjusted *p* < 0.05) identified 1888 genes that were significantly upregulated in GQF‐treated mice compared with HFD‐fed mice (Figure 7A). Gene set enrichment analysis (GSEA) and over‐representation analysis of all statistically significant DEGs revealed that Gene Ontology (GO) Biological Pathway terms pertaining to synaptic transmission and neural plasticity were significantly enriched (Figure 7B,C). Specifically, GQF selectively increased the expressions of *Stx17*, *Sytl2*, *Ntrk2*, *Grik3*, *Grid2*, *Nexmif*, *Nrg1* and *Sytl5* (Figure 7A). Consistent with the effects of GQF, GO enrichment analysis revealed that FA treatment also promoted synaptic transmission and neuronal plasticity in obese mice (Figure S7C). In particular, genes associated with synaptic plasticity (*Syt10*, *Stxbp1*, *Grin3a*, *Synpo2*, and *Nexmif*) and neuronal growth (*Ngfr*, *Ntrk2*, *Crebrf*, *Gfra1*, and *Glg1*), which were markedly suppressed in obese mice, were significantly upregulated following FA treatment (Figure S7B and Figure 7D). GSEA further demonstrated that dendrite morphogenesis and synaptic membrane component pathways were broadly inhibited in the hippocampus of obese mice, whereas both GQF and FA treatments effectively reversed these impairments (Figure 7E–G and Figure S7D,E). These transcriptomic findings were highly consistent with the observed biochemical and structural outcomes. Collectively, these results indicate that FA mediates the beneficial effects of GQF on learning and memory by enhancing synaptic function, which is accompanied by the upregulation of hippocampal synaptic transmission‐related genes.

**FIGURE 7:**
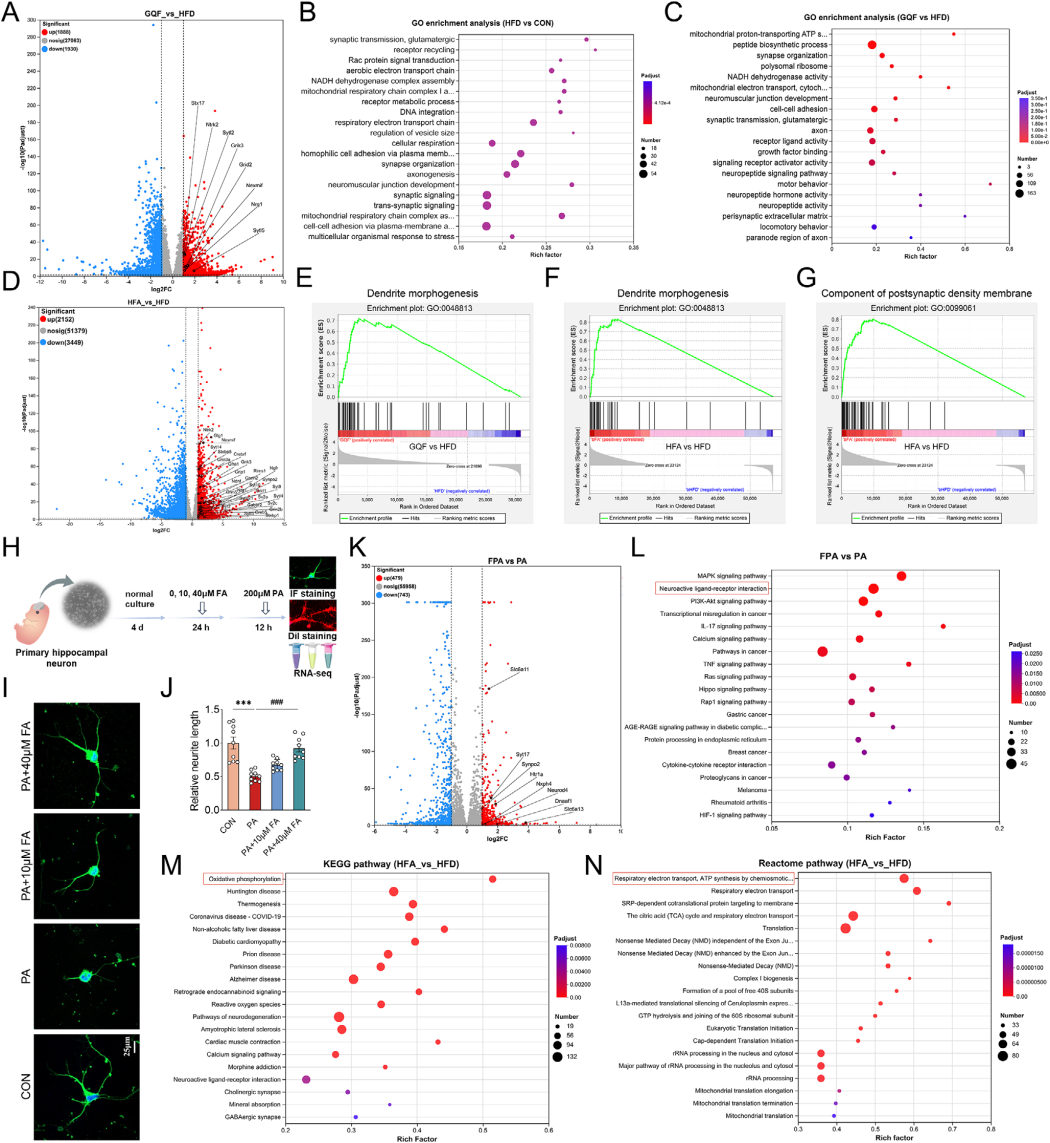
FA mediates GQF‐induced improvement of synaptic plasticity by regulating hippocampal synaptic transmission‐related genes. (A) Volcano plot displaying 3818 differentially expressed genes (DEGs, FDR‐p < 0.05) in the hippocampus from GQF vs. HFD group (GQF refers to the HGQF group, *n* = 6). Genes related to synaptic transmission and neuroplasticity are denoted. (B−C) GO enrichment analysis of differential biological functions in hippocampus of HFD vs. CON group (B), and GQF vs. HFD group (C). (D) Volcano plot displaying 5601 differentially expressed genes (DEGs, FDR‐p < 0.05) in the hippocampus from HFA vs. HFD group (*n* = 6). Genes related to synaptic transmission and neuroplasticity are denoted. (E−G) Representative GSEA of differential biological processes triggered by GQF or HFA. (H−J) Representative βIII‐Tubulin staining images of primary hippocampal neurons treated with PA (200 µM) alone or FA pre‐treatment (10, 40 µM, I) and quantification of neurite length (J, *n* = 9 neurons per group). Scale bar, 25 µm. (K) Volcano plot displaying 1222 differentially expressed genes (DEGs, FDR‐p < 0.05) in the primary hippocampal neurons from FPA vs. PA group (*n* = 3). Genes related to synaptic transmission and neuroplasticity are denoted. (L) KEGG pathway analysis of differential biological processes in the primary hippocampal neurons from FPA vs. PA group (*n* = 3). (M,N) KEGG (M) and Reactome (N) pathway analysis of differential biological processes in hippocampus of HFA vs. HFD group (*n* = 6). Data are presented as the means ± SEM. ^***^ *p* < 0.001 vs. CON group. ^###^ *p* < 0.001 vs. PA group.

To determine whether FA directly influences synaptic plasticity, primary hippocampal neurons derived from fetal mice were cultured in vitro. Palmitic acid (PA) treatment markedly inhibited neurite outgrowth and differentiation of primary hippocampal neurons, consistent with the in vivo findings. In contrast, FA pretreatment (10 or 40 µM) significantly increased total neurite length and number (Figure 7H–J). Moreover, FA markedly alleviated PA‐induced impairments in dendritic complexity (Figure S7F). These findings demonstrate that FA preserves synaptic plasticity by modulating neuronal dendritic architecture. To further explore the underlying mechanisms, RNA sequencing was performed on FA‐treated primary hippocampal neurons. Compared with PA‐treated neurons, FA pretreatment resulted in 1,222 DEGs (FDR‐adjusted *p* < 0.05) (Figure 7K). Notably, genes associated with synaptic transmission (*Htr1a*, *Syt17*, *Slc6a13*, *Synpo2*, and *Slc6a11*) and neurodevelopment (*Dnaaf1*, *Nxph4*, and *Neurod4*), which were downregulated by PA treatment, were significantly restored by FA (Figure S7G and Figure 7K). These changes underscore the critical role of synaptic transmission and neuronal plasticity in FA‐mediated neuroprotection. Consistently, FA treatment restored neuroactive ligand–receptor interaction function (Figure 7L and Figure S7H). GSEA further revealed that FA enhanced synaptic function while suppressing pathways of neurodegeneration, supporting a neuroprotective role for FA in regulating synaptic plasticity (Figure S7I–K). These in vitro findings closely mirror the in vivo results. Taken together, these results demonstrate that FA mediates the neuroprotective effects of GQF by regulating neuronal growth and synaptic plasticity, which in turn alleviates obesity‐associated cognitive impairment.

### 2.8 FA Promotes Neuronal Growth and Synaptic Plasticity by Activating Mitophagy Pathway Associated with PINK1/Parkin in the Hippocampus

Based on transcriptomic pathway enrichment analyses, we further investigated the cellular signaling pathways and downstream molecular mechanisms through which FA improves synaptic function. KEGG and Reactome pathway enrichment analyses revealed that FA treatment predominantly enriched pathways associated with mitochondrial function in the hippocampus, including oxidative phosphorylation (OXPHOS), ATP synthesis, and respiratory electron transport (Figure 7M,N). These findings were further corroborated by pathway enrichment analyses of hippocampal transcriptomes from GQF‐treated mice (Figure S7L,M), which similarly identified OXPHOS and mitochondrial functional regulation as prominent features. Mitochondria supply ATP required for synaptic activity through OXPHOS, thereby ensuring efficient synaptic transmission [28]. Conversely, mitochondrial dysfunction leads to impaired synaptic energy supply, disruption of calcium homeostasis, and excessive accumulation of reactive oxygen species (ROS), ultimately resulting in synaptic abnormalities and the development of neurological disorders [29]. Therefore, based on the current experimental evidence, we propose that the FA‐mediated improvement of synaptic dysfunction following GQF intervention is closely linked to the regulation of mitochondrial function.

Given that chronic HFD consumption induces mitochondrial structural and functional impairment, we first examined mitochondrial ultrastructure in the hippocampus using transmission electron microscopy. In obese mice, the proportion of abnormal mitochondria was markedly increased, characterized by pronounced mitochondrial deformation, disorganized or absent cristae, and a darker matrix. Notably, FA intervention significantly reduced the proportion of abnormal mitochondria and led to the appearance of abundant mitophagosomes in hippocampal neurons (Figure 8A,B). Similar mitochondrial alterations were observed in the hippocampus of GQF‐treated mice (Figure S8A,B). Consistently, RNA‐seq analysis revealed that FA treatment prevented the obesity‐induced downregulation of autophagy‐related genes, including *Atg2b*, *Epg5*, and *Soga1* (Figure 8C and Figure S8C). To further assess mitophagy, immunofluorescence co‐staining was performed using cytochrome c (CYCS), a mitochondrial intermembrane space protein, to label mitochondria, and microtubule‐associated protein light chain 3 (LC3) to label autophagosomes. In the hippocampus of obese mice, LC3–CYCS colocalization was markedly reduced, indicating impaired hippocampal mitophagy. In contrast, FA treatment significantly increased LC3 colocalization with CYCS (Figure 8D,E). Western blot analysis further confirmed these findings, showing that FA treatment increased LC3 expression while reducing p62 levels in the hippocampus compared with HFD‐fed mice (Figure 8F–H). These effects were consistent with those observed in GQF‐treated mice (Figure S8D–H). The PINK1/Parkin signaling pathway is a central regulator of mitophagy [30, 31]. Accordingly, we examined the expression of key components of this pathway. Consistent with previous reports, protein levels of PINK1 and Parkin were reduced in the hippocampus of obese mice. Importantly, both FA and GQF treatments significantly upregulated the expression of PINK1 and Parkin (Figure 8I–N and Figure S8I–N).

**FIGURE 8:**
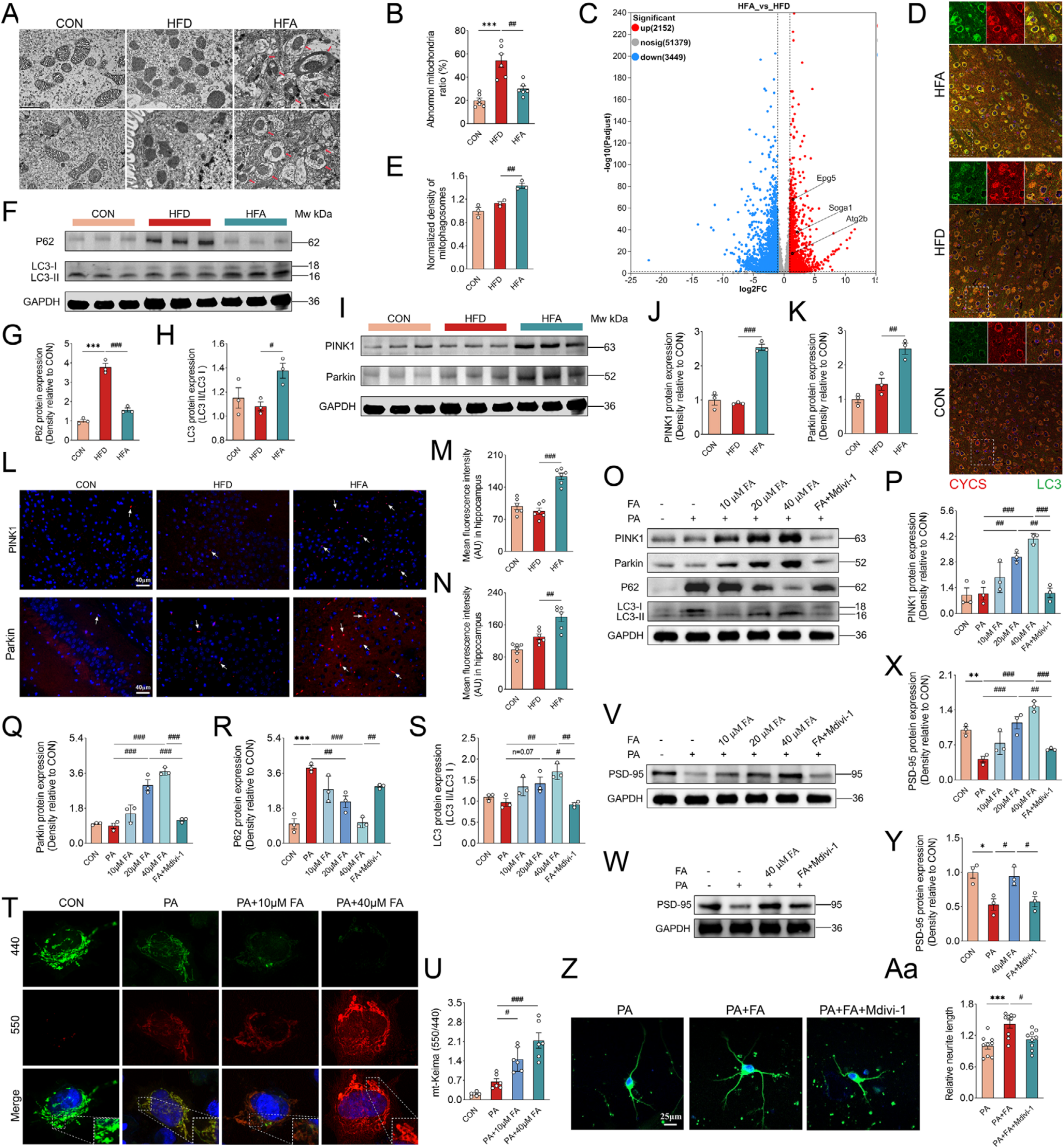
FA promotes neuronal growth and synaptic plasticity by activating hippocampal mitophagy associated with PINK1/Parkin pathway. (A) Representative images of hippocampal mitochondria ultrastructure by transmission electron microscope. Scale bar, 1 µm. The arrowheads stand for the representative mitophagosomes. (B) Abnormal mitochondria ratio (*n* = 6 slices from 3 mice). (C) Volcano plot displaying 5601 differentially expressed genes (DEGs, FDR‐p < 0.05) in the hippocampus from HFA vs. HFD group (*n* = 6). Genes related to mitophagy are denoted. (D,E) Representative confocal microscopy images (×20) and quantitative analysis showing enhanced mitophagosome accumulation in the hippocampal sections of mice treated with FA. The density of mitophagosomes in the hippocampus was quantified and normalized to that of control mice supplemented with normal saline (*n* = 3). (F−H) Protein expression levels of hippocampal P62 and LC3 in mice (*n* = 3). (I−K) Protein expression levels of hippocampal PINK1 and Parkin in mice (*n* = 3). Western Blots in Figure F and I originated from the same batch of mouse tissues, exhibiting consistent GAPDH loading control bands. (L) Representative immunofluorescence staining images of PINK1 and Parkin from hippocampal sections. Scale bar, 40 µm. The arrowheads stand for the representative PINK1 or Parkin. (M−N) Quantification of the PINK1 (M) and Parkin (N) area based on immunofluorescence staining sections by ImageJ software in the hippocampus (*n* = 6 slices from 3 mice). (O−S) Protein expression levels of PINK1, Parkin, P62, and LC3 in 300 µM PA‐treated SH‐SY5Y cells with FA (0, 10, 20, 40 µM) or 40 µM FA + 25 µM Mdivi‐1 (*n* = 3). (T−U) Mitophagy detected using pMDV‐mCMV‐mt‐Keima transfection. After transfection with adenovirus, SH‐SY5Y cells were treated with 300 µM PA, 300 µM PA + 10 µM FA, or 300 µM PA + 40 µM FA, respectively, then cells were observed by confocal microscope (×100 oil). Mitophagy level is indicated by the ratio of red to green fluorescence intensity (*n* = 6 slices from 3 independent repeats). (V, X) Protein expression levels of PSD‐95 in 300 µM PA‐treated SH‐SY5Y cells with FA (0, 10, 20, 40 µM) or 40 µM FA + 25 µM Mdivi‐1 (*n* = 3). Western Blots in Figure O and V were prepared from a single batch of cultured cell samples, with matching GAPDH loading control bands. (W,Y) Protein expression levels of PSD‐95 in 200 µM PA‐treated primary hippocampal neurons with FA (0, 40 µM) or 40 µM FA + 25 µM Mdivi‐1 (*n* = 3). (Z, Aa) Representative βIII‐Tubulin staining images of primary hippocampal neurons treated with PA (200 µM) alone, FA pre‐treatment (40 µM), or Mdivi‐1 (25 µM) + FA (40 µM) pre‐treatment and quantification of neurite length (Aa, *n* = 9 neurons from 3 independent repeats). Scale bar, 25 µm. Data are presented as the means ± SEM. (A−N) ^***^ *p* < 0.001 vs. CON group. ^#^ *p* < 0.05, ^##^ *p* < 0.01, and ^###^ *p* < 0.001 vs. HFD group. (O−Y) ^*^ *p* < 0.05, ^**^ *p* < 0.01, and ^***^ *p* < 0.001 vs. CON group. ^#^ *p* < 0.05, ^##^ *p* < 0.01, and ^###^ *p* < 0.001 vs. FA (10, 20, or 40 µM) group. (Aa) ^***^ *p* < 0.001 vs. PA group. ^#^ *p* < 0.05 vs. PA + FA group.

We next performed in vitro experiments using SH‐SY5Y cells and primary hippocampal neurons. FA pretreatment dose‐dependently attenuated PA‐induced cytotoxicity (Figure S8O–Q). FA markedly alleviated PA‐induced mitochondrial dysfunction in SH‐SY5Y neuronal cells, as evidenced by reduced intracellular ROS levels, restoration of Ca^2^^+^ homeostasis, and increased mitochondrial membrane potential and ATP production. Notably, these protective effects of FA on mitochondrial function were significantly blunted by treatment with Mdivi‐1, a mitophagy inhibitor (Figure S8R–V). With prolonged PA exposure, expression of the mitophagy markers PINK1, Parkin, and LC3 in SH‐SY5Y cells initially increased and subsequently declined, whereas p62 exhibited the opposite trend (Figure S8W–Z and S8Aa). These findings suggest that early PA exposure transiently activates mitophagy as a compensatory self‐protective response; however, sustained PA treatment suppresses mitophagic activity, leading to the accumulation of damaged mitochondria and subsequent neuronal dysfunction. Importantly, FA treatment restored the expression of mitophagy‐related proteins (PINK1, Parkin, and LC3) and reduced p62 levels, effects that were abolished by Mdivi‐1 treatment (Figure 8O–S). To directly assess mitophagy flux, SH‐SY5Y cells were transfected with the pMDV‐mCMV‐mt‐Keima adenoviral reporter and subsequently exposed to FA followed by PA treatment. FA pretreatment significantly increased the mt‐Keima fluorescence intensity ratio (550/440), indicating enhanced mitophagic flux in SH‐SY5Y cells (Figure 8T–U). In addition, FA treatment rescued PA‐induced reductions in PSD‐95 expression in both SH‐SY5Y cells and primary hippocampal neurons and significantly increased neurite length in primary hippocampal neurons. Importantly, inhibition of mitophagy with Mdivi‐1 attenuated the protective effects of FA on synaptic dysfunction (Figure 8V–Z and Figure 8Aa). Collectively, these findings suggest that FA improves mitochondrial function and protects synaptic plasticity by activating hippocampal mitophagy, which is accompanied by the upregulation of PINK1/Parkin signaling pathway.

In summary, this study elucidates a previously unrecognized molecular mechanism by which GQF ameliorates metabolic‐associated cognitive impairment. This mechanism depends on the microbial release of FA by specific gut bacteria, *R. hominis* and *R. intestinalis*, which activates hippocampal mitophagy to restore mitochondrial function and, in turn, preserve synaptic integrity.

## 3 Discussion

A growing body of evidence has highlighted the effectiveness of whole‐grain diets in ameliorating obesity and obesity‐associated cognitive decline, effects that are often attributed, at least in part, to alterations in the gut microbiota. However, whether GQF can alleviate obesity‐related cognitive deficits and the extent to which the gut microbiota contributes to these effects have remained unclear. Here, our study not only demonstrates the beneficial effects of GQF on HFD‐induced cognitive impairment but also elucidates the underlying mechanisms via a specific gut microbiota–metabolite–brain axis. Using multi‐omics combined analysis, we identified the key bioactive mediators, core gut microbiota, bacterial enzymes, and brain response genes involved in the improvement of cognitive dysfunction by GQF. Furthermore, research approaches including FMT, functional validation of core bacteria, supplementation of bioactive mediators, and specific blockade of target molecules were utilized to elucidate the causal mechanisms by which GQF alleviates learning and memory dysfunction through the gut microbiota–FA–brain axis in obese mice. Finally, we demonstrated that GQF‐enriched *R. hominis* and *R. intestinalis* promote the in vivo release of bound FA from GQF via FAE activity, increase FA tissue distribution, and thereby target the FA–mitophagy–synapse signaling axis to ameliorate cognitive impairment.

Dietary composition represents one of the most common and powerful environmental factors influencing metabolic diseases and human health [32]. Our previous work demonstrated that, compared with QF, GQF contains significantly higher levels of dietary fiber, amino acids, and polyphenols, as well as enhanced antioxidant capacity, suggesting substantial health‐promoting potential [19]. However, reports describing the biological functions of GQF remain limited, and most existing studies have been conducted in vitro. In the present study, we show that GQF more effectively ameliorates obesity phenotypes and learning and memory impairments in HFD‐fed mice than QF, a finding that, to our knowledge, has not been previously reported. Consistent increases in synaptic proteins, including BDNF, PSD‐95, and SYP, together with enhanced dendritic spine density and improved synaptic ultrastructure, further support the beneficial effects of GQF on synaptic function. Nevertheless, additional studies employing electrophysiological approaches, such as paired‐pulse facilitation (PPF) and long‐term potentiation (LTP), are required to directly assess the impact of GQF on synaptic transmission in obese mice, which will provide more direct evidence for GQF‐mediated synaptic plasticity improvement.

Given the well‐established link among gut microbiota, dietary interventions, and cognitive function, together with the prominent regulatory effect of GQF on gut microbial composition, we further explored the microbial mechanism underlying GQF‐mediated cognitive improvement in obese mice. Our FMT results demonstrated that transplantation of GQF‐modulated gut microbiota alone failed to fully recapitulate the cognitive benefits of GQF intervention, whereas combined treatment with FMT and dietary GQF supplementation markedly improved gut microbial diversity and cognitive performance. These findings strongly indicate that the neuroprotective efficacy of GQF exhibits typical microbiota‐substrate co‐dependent characteristics. Specifically, gut microbiota does not directly exert comprehensive neuroprotective effects, and its core functional role is to decompose and liberate bound‐state FA and other active metabolites from GQF dietary substrates. Only when functional intestinal flora is matched with sufficient dietary substrates can bioactive metabolites be efficiently generated to mediate subsequent neuroprotective responses. Accumulated evidence has demonstrated that dietary components modulate host physiological status and disease susceptibility by driving microbiota‐dependent production of small bioactive molecules [13]. Distinct from de novo microbial fermentation products, dietary bioactive compounds enzymatically liberated by gut microbiota constitute crucial mediators of host beneficial effects. Consistent with this principle, our results support a vital functional interplay between GQF and gut microbiota that underpins the cognitive‐protective outcomes of GQF treatment. This mechanistic model is further validated by our subsequent experimental evidence showing that GQF‐enriched *Roseburia* serves as a key functional bacterium capable of releasing bioactive free FA from GQF substrates.

During grain germination, the activation of endogenous enzymes induces profound metabolic remodeling of chemical constituents [33, 34]. In the present study, we observed a marked upregulation of the phenylalanine metabolism pathway following quinoa germination, consistent with previous reports [35]. Phenylalanine metabolism represents one of the most critical secondary metabolic pathways in plants, as compounds containing a phenylpropanoid backbone are directly or indirectly derived from this pathway [36]. Notably, secondary metabolites such as phenolic acids, flavonoids, and lignans—key contributors to nutritional and health‐promoting properties—originate from phenylalanine metabolism. In our study, polyphenols accounted for as much as 24.34% of the metabolites upregulated in GQF compared with QF, among which the cereal‐characteristic polyphenol FA exhibited particularly pronounced changes. Based on these observations, we propose that FA represents a key material basis underlying the potent biological activity of GQF. However, according to existing literature, FA in cereal grains is predominantly esterified and cross‐linked within the plant cell wall matrix to form feruloylated oligosaccharides [24, 37]. Consistently, our results indicate that FA in GQF exists mainly in a bound form. Covalently bound FA is resistant to endogenous digestive enzymes and is neither hydrolyzed nor absorbed in the small intestine, thereby limiting its bioavailability and biological efficacy [38, 39]. These considerations led us to hypothesize that specific gut microorganisms are required to efficiently liberate large quantities of bound FA, enabling GQF to exert its pronounced cognitive benefits—a hypothesis that was validated by our subsequent experiments identifying *Roseburia* as the key FA‐liberating gut bacteria.

Gut microorganisms modify a wide range of dietary substrates through the coordinated action of specific genetic repertoires and enzymes, and these metabolic processes are closely linked to health benefits as well as disease susceptibility [40]. Because the evolution and acquisition of specific CAZymes confer competitive advantages to certain bacteria, human gut microbes devote a substantial proportion of their genomes to CAZyme‐encoding genes [41, 42, 43]. Representative microbial phyla, particularly Bacteroidetes and Firmicutes, have evolved sophisticated strategies to degrade complex polysaccharides in the distal gastrointestinal tract. Notably, several Bacteroidetes members, including *Bacteroides intestinalis*, *B. thetaiotaomicron*, and *B. ovatus*, have been shown to encode CEs that facilitate the degradation of complex polysaccharides and liberate bioactive compounds such as phenolic acids from side chains [44]. More recently, human gut Firmicutes members, including *Roseburia* and *Faecalibacterium*, have been reported to harbor polysaccharide utilization loci (PULs) encoding mannan‐specific esterases [45]. Despite these advances, the identity of colonic microorganisms responsible for FA release and the associated fermentation mechanisms in the human gut remain poorly understood. FAE has been identified as one of the key enzymes involved in the liberation of complex, bound FA. A recent study reported that the Bacteroidetes members *B. intestinalis, B. eggerthii*, *B. oleiciplenus*, and *B. cellulosilyticus* possess PULs encoding esterases capable of depolymerizing FA [44]. In the present study, we found that the fecal metagenomes of GQF‐treated mice were enriched in CAZyme‐encoding genes, supporting enhanced degradation and metabolism of GQF in vivo. Importantly, another striking finding of this work is the identification of the Firmicutes members *R. hominis* and *R. intestinalis* as characteristic taxa enriched following GQF intervention, both harboring genes encoding FAE. Functional expression of FAE in *R. hominis* and *R. intestinalis* was confirmed in vitro and further validated by whole‐genome sequence analysis. In vivo, gavage with *R. hominis* significantly increased fecal FAE activity. Subsequent in vitro and in vivo experiments established the capacity of these bacteria to release bound FA from GQF, providing a foundation for further exploration and utilization of Firmicutes‐mediated phenolic metabolism. The cross‐linked structures formed by FA in GQF and the organization of FAE‐encoding PULs in *Roseburia* warrant further investigation. Overall, our findings demonstrate that GQF intake selectively enriches *Roseburia* in the gut, which in turn promotes microbial liberation of FA from dietary substrates, increasing its distribution in peripheral circulation and the brain. This process likely contributes to the neuroprotective effects of GQF. To facilitate clinical translation, future studies should evaluate the relationship between fecal FA levels and *Roseburia* abundance in human subjects following GQF consumption.

Free FA has been shown to cross the blood‐brain barrier and to exert diverse biological activities [23, 46]. We therefore hypothesized that FA released by gut microorganisms mediates the neuroprotective effects of GQF in obese mice. As anticipated, dietary FA administered at higher doses—corresponding to the combined amount of free and bound FA present in GQF—activated hippocampal mitophagy and modulated mitochondrial function, thereby ultimately preserving synaptic plasticity, an effect accompanied by the upregulation of PINK1/Parkin signaling pathway. These effects closely mirrored those observed following GQF intervention. Previous studies have demonstrated that chronic HFD consumption induces defects in PINK1/Parkin‐mediated mitophagy, leading to mitochondrial dysfunction and synaptic damage [47, 48, 49]. In addition, FA has been reported to alleviate neuronal degeneration, potentially through stimulation of autophagic processes [50]. Together with recent evidence, these findings suggest that mitophagy impairment may accelerate cognitive decline through a dual mechanism involving mitochondrial dysfunction and synaptic abnormalities. Collectively, these observations underscore a critical role for mitophagy in maintaining synaptic plasticity homeostasis and identify it as a key regulatory node in metabolic‐associated cognitive impairment. As such, mitophagy represents a promising therapeutic target for the treatment of metabolic‐related cognitive deficits in the foreseeable future.

The present study has some limitations that should be noted. In investigating the role of *Roseburia*‐derived FAE in releasing bound FA from GQF, this study employed methods such as gene annotation, enzymatic activity assays, in vitro fermentation, and in vivo colonization. Future research should incorporate *Roseburia* strains with *Fae* gene knockout to more deeply explore the release of FA mediated by *Roseburia*‐derived FAE and its subsequent role in GQF‐elicited neuroprotection. Additionally, although our results demonstrate that FA modulates hippocampal mitophagy with a concomitant upregulation of the PINK1/Parkin signaling pathway, future studies employing conditional *Pink1* knockout mouse models will be necessary to definitively establish the causal role of PINK1/Parkin signaling in FA‐induced cognitive improvement in obese mice. Furthermore, future studies employing proteomics, structural biochemistry, and bacterial genomics to investigate the detailed degradation mechanism of the cross‐linked FA structure in GQF will be of great significance for elucidating the biotransformation of dietary polyphenols. Finally, the beneficial effects of GQF on obesity and brain health observed in this study require further validation in well‐designed clinical investigations involving human subjects to support translational relevance and potential clinical application.

Overall, this study provides compelling evidence supporting GQF intervention as a novel and effective approach for improving obesity‐related cognitive impairment through the gut microbiota–FA–mitophagy axis, thereby addressing a critical challenge in the field of metabolism‐related disorders. In the present study, we demonstrate for the first time that GQF is more effective than QF in preventing obesity‐induced cognitive impairment. These benefits are associated with a GQF‐driven increase in the abundance of *Roseburia* and enrichment of genes encoding FAE. Notably, single gut microbiota intervention cannot exert equivalent effects due to the absence of fermentable substrates, which further confirms that the metabolic activation of dietary polyphenols by intestinal flora is a prerequisite for exerting downstream physiological functions. More importantly, within the current experimental model, this study provides the first evidence identifying *R. hominis* and *R. intestinalis* as key gut bacteria carrying FAE‐encoding genes and possessing the capacity to release bound FA from GQF, as validated in both in vivo and in vitro assays. We further demonstrate that FA, as a key polyphenol enriched in GQF, is liberated by intestinal *Roseburia* and subsequently participates in the regulation of hippocampal mitophagy and mitochondrial function, thereby maintaining synaptic integrity and normal cognitive function. These findings highlight the critical role of the gut microbiota‑substrate co‑dependent effects in dietary nutritional interventions and provide mechanistic insights into novel therapeutic strategies for metabolic‐associated neurodegenerative diseases.

## 4 Methods

### 4.1 Mice

Male C57BL/6J mice (6 weeks old) were purchased from Beijing Vital River Laboratory Animal Technology Co., Ltd. Mice were housed under standard conditions at the Laboratory Animal Center of Northwest A&F University. All animal care and experiments were carried out in strict accordance with the recommendations of the Guide for the Care and Use of Laboratory Animals of the Ministry of Health (China). All procedures were approved by the animal ethics committee of Northwest A&F University (Approval No. IACUC2025‐0101).

### 4.2 Bacterial Culture

The bacterial strains *Roseburia intestinalis* and *Roseburia hominis* were purchased from the German Collection of Microorganisms and Cell Cultures (DSMZ). They were routinely cultured under anaerobic conditions (85% N_2_, 5% CO_2_, 10% H_2_) at 37°C in DSMZ medium 1611.

### 4.3 Primary Hippocampal Neuron Isolation and Culture

Primary hippocampal neurons were isolated from E16.5 mouse embryos. Following euthanasia, embryonic brains were collected in cold Hanks’ Balanced Salt Solution (HBSS). Under a stereomicroscope, the hippocampi were microdissected, minced thoroughly with fine scissors, and digested with 2 mg/mL papain at 37°C for 30 min. After centrifugation at 1000 × g for 5 min and resuspension, cells were counted and plated on pre‐coated culture dishes or plates for subsequent experiments.

### 4.4 SH‐SY5Y Culture

SH‐SY5Y cells were obtained from the Kunming Cell Bank (KCB) of the Chinese Academy of Sciences. Cells were cultured in RPMI 1640 medium supplemented with 10% fetal bovine serum (FBS), 1% penicillin‐streptomycin, 1% non‐essential amino acids, 1% L‐glutamine, and 1% sodium pyruvate at 37°C with 5% CO_2_.

### 4.5 Preparation of Germinated Quinoa

Prior to germination, quinoa seeds were surface‐sterilized with 4% sodium hypochlorite (5 min) and rinsed thoroughly with deionized water. After a 6‐h hydration period, the seeds were placed on moist filter paper in perforated trays and germinated in the dark within a controlled climate chamber maintained at 25°C and 95% relative humidity. After 48 h of germination, the samples were then freeze‐dried, ground to pass through a 100‐mesh sieve, and stored at −20°C as germinated quinoa flour (GQF) until further analysis. Non‐germinated quinoa flour (QF) was prepared from seeds collected at 0 h of germination. The nutritional components of GQF and QF was shown in Table S3.

### 4.6 Animal Experiments

#### 4.6.1 Animal Experiment 1

Mice were randomly divided to six groups (*n* = 12): (1) Control group (CON; standard diet D12450J, 10% kcal from fat), (2) High‐fat diet group (HFD; D12492, 60% kcal from fat), (3) LQF group, fed the HFD supplemented with 10% QF, (4) HQF group, fed the HFD supplemented with 30% QF, (5) LGQF group, fed the HFD supplemented with 10% GQF, (6) HGQF group, fed the HFD supplemented with 30% GQF. The QF or GQF replaced corresponding amounts of starch and cellulose in the HFD. The experimental diet was obtained from Jiangsu Xietong Pharmaceutical Bio‐Engineering Co., Ltd. and the detailed composition listed in Tables S4 and S5. Except for the CON group, the diets for the other groups were isocaloric. Following a 10‐week intervention, behavioral assessments were performed.

#### 4.6.2 Animal Experiment 2

Mice were randomly divided to three donor groups (*n* = 8) and fed for 10 weeks: (1) CON donors: standard diet, (2) HFD donors: HFD, (3) HGQF donors: HFD supplemented with 30% GQF. A separate cohort mice served as recipients. After 2‐week antibiotic cocktail to deplete gut microbiota, recipients were randomly divided into four groups (*n* = 10) and underwent microbiota reconstitution via oral gavage of corresponding donor fecal suspensions for 3 consecutive days: (1) FCON: received CON donor microbiota, then maintained on a standard diet for 10 weeks, (2) FHFD: received HFD donor microbiota, then maintained on a HFD for 10 weeks, (3) FGH: received HGQF donor microbiota, then maintained on a HFD for 10 weeks, (4) FGQ: received HGQF donor microbiota, then maintained on the HGQF diet (HFD + 30% GQF) for 10 weeks. During the 10‐week intervention, recipients received gavages of the corresponding donor fecal supernatant twice weekly. All mice were housed under germ‐free conditions. Recipient mice were fed a standard diet during antibiotic treatment and the 3‐day microbiota reconstitution period.

#### 4.6.3 Animal Experiment 3

To assess ferulic acid (FA) release from GQF by *R. hominis*, mice were depleted of gut microbiota via a 2‐week antibiotic cocktail treatment. They were then divided into four groups (n = 8) and treated for 2 weeks (every‐other‐day gavage) as follows: (1) AHFD: 0.2 mL sterile PBS, followed by HFD for 3 weeks, (2) AGQF: 0.2 mL sterile PBS, followed by a HFD supplemented with 30% GQF for 3 weeks, (3) ARH: 0.2 mL of *R. hominis* suspension (1 × 10^9^ CFU/mL), followed by a HFD for 3 weeks, (4) ARG: 0.2 mL of *R. hominis* suspension (1 × 10^9^ CFU/mL), followed by a HFD supplemented with 30% GQF for 3 weeks. *R. hominis* colonization was verified by RT‐qPCR. All mice received a standard diet during antibiotic treatment and the 2‐week gavage period.

#### 4.6.4 Animal Experiment 4

To specifically investigate the effects of FA released from GQF, independently of other GQF components, a direct FA supplementation study was conducted. Mice were randomly divided to four groups (*n* = 12) and treated for 10 weeks as follows: (1) CON: standard diet + daily gavage of saline, (2) HFD: HFD + daily gavage of saline, (3) LFA: HFD + daily gavage of saline containing FA, with a administer dose of 10 mg/kg/day, (4) HFA: HFD + daily gavage of saline containing FA, with a administer dose of at 40 mg/kg/day. Behavioral tests were performed at the end of the 10‐week intervention period.

### 4.7 Fecal Microbiome Transplantation

Donor fecal supernatant was prepared aseptically by homogenizing fresh feces in PBS (10 mL/g), followed by low‐speed centrifugation (500 × g, 3 min). Recipient mice were pre‐treated for 2 weeks with a broad‐spectrum antibiotic cocktail (metronidazole, ampicillin, streptomycin, neomycin, and vancomycin in sterile drinking water) to deplete gut microbiota. Depletion was confirmed by culture on BHI agar and RT‐qPCR. For microbiota reconstitution, recipient mice were gavaged with 0.2 mL of the respective donor supernatant for 3 consecutive days, followed by twice‐weekly gavages treatment during the subsequent dietary intervention period.

### 4.8 Behavioral Testing

All behavioral tests were conducted at the end of the experimental period. On each test day, mice were brought to the testing room and allowed to acclimate for at least 1 h. All equipment was cleaned with 75% ethanol between trials.

#### 4.8.1 Open Field Test

Mice were gently placed in the center of an open field arena (40 × 40 × 40 cm^3^) and allowed to explore freely for 5 min. Total distance traveled and distance moved in the center zone were recorded and analyzed using SuperMaze software (Shanghai Xinruan Information Technology Co., Ltd., China).

#### 4.8.2 Y‐Maze Test

The Y‐maze apparatus consisted of three identical opaque arms (35 × 35 × 35 cm^3^) positioned at 120° angles to each other. Mice were placed in the central area and allowed to explore freely for 5 min. An arm entry was recorded when all four paws entered an arm. A spontaneous alternation was defined as consecutive entries into three different arms. The total number of arm entries and the number of spontaneous alternations were recorded. The percentage of spontaneous alternation was calculated as: [number of spontaneous alternations / (total arm entries − 2)] × 100.

#### 4.8.3 Novel Object Recognition Test

The test was conducted over 3 days and comprised three phases: habituation, training, and testing. On day 1 (habituation), mice freely explored the empty open field arena for 5 min. On day 2 (training), two identical objects were placed in the arena, and mice were allowed to explore for 5 min. After a 24‐h interval, one familiar object was replaced with a novel object on day 3 (testing), and the mice explored again for 5 min. Object exploration was defined as sniffing, licking, or touching the object with the nose or forepaws when the mouse's head was within 2 cm of the object. A discrimination index was calculated to assess novel object preference: Discrimination index = (time exploring the novel object − time exploring the familiar object) / total exploration time.

#### 4.8.4 Barnes Maze Test

The apparatus consisted of a circular platform (90 cm in diameter) with 20 evenly spaced holes (5 cm in diameter) around the perimeter. An escape box was placed under one designated target hole. The test lasted 6 days and included three phases: habituation, training, and probe trial. On day 1 (habituation), mice were first placed in the escape box for 1 min, then allowed to explore the maze for 2 min, and finally returned to the escape box for another 1 min. During the 4‐day training phase (days 2–5), mice were placed under an opaque start box in the center of the platform for 30 s, then allowed to explore for up to 5 min to find the escape box, where they remained for 1 min. If a mouse entered the escape box before 5 min, the trial ended, and the mouse stayed in the box for 1 min. On day 6 (probe trial), the escape box was removed, and mice explored the maze for 5 min. The escape latency to reach the target hole and the entries in the probe zone during the probe trial were recorded.

### 4.9 Cell Experiments

#### 4.9.1 Cell Viability Assay

Cell viability of SH‐SY5Y cells treated with palmitic acid (PA) and ferulic acid FA was assessed using a CCK‐8 assay. Briefly, SH‐SY5Y cells in the logarithmic growth phase were seeded into 96‐well plates at a density of 1 × 10^4^ cells per well. After attachment, cells were pre‐treated with various concentrations of FA for 24 h, followed by exposure to different concentrations of PA for 12 h. Control wells received medium containing 0.4% BSA. Cell viability was then measured according to the manufacturer's protocol.

#### 4.9.2 Cell Treatment Experiments

After 4 days in vitro (DIV4), primary hippocampal neurons were treated with 0, 10, or 40 µM FA for 24 h, followed by incubation with 200 µM PA for 12 h (control: 0.4% BSA). Neurite length and dendritic complexity were subsequently analyzed.

After attachment, SH‐SY5Y cells were cultured conventionally for 24 h. Mitochondrial autophagy proteins were detected after incubating the cells with 300 µM PA for 0, 3, 6, 9, or 12 h, respectively.

To test FA's protective effects, cells were pre‐treated with 0, 10, 20, or 40 µM FA for 24 h, followed by co‐incubation with 300 µM (or 200 µM) PA for 12 h before analysis.

To inhibit mitophagy, cells were pre‐treated with a selected concentration of FA for 24 h, followed by incubation with 25 µM Mdivi‐1 for 3 h prior to PA exposure. Analysis was performed 12 h later.

#### 4.9.3 Detection of Mitochondrial Function

Intracellular ROS levels, calcium ion concentration, and mitochondrial membrane potential (ΔΨm) were measured using specific commercial assay kits according to the manufacturers' instructions, with images acquired by confocal microscopy. Cellular ATP levels were quantified using an ATP assay kit and a multi‐mode microplate reader.

#### 4.9.4 Adenovirus Transfection and Analysis of Mitophagic Flux

To monitor mitophagic flux, SH‐SY5Y cells were transfected with an adenovirus encoding mitochondria‐targeted Keima (pMDV‐mCMV‐mt‐Keima, OBiO Technology). mt‐Keima fluorescence was visualized using a laser scanning confocal microscope (LSM 980, Zeiss, Germany), allowing for the quantification of mitochondria delivered to lysosomes.

#### 4.9.5 Dil Staining of Primary Hippocampal Neurons

Fixed and washed primary hippocampal neurons were incubated with the lipophilic fluorescent dye Dil at room temperature for 15 min to label neuronal membranes. After PBS washes, nuclei were counterstained with DAPI. Images were captured using a confocal microscope.

### 4.10 16S rRNA Microbiome Sequencing

Following the intervention, fecal samples were collected from mice in each group under sterile conditions and immediately stored at −80 °C until analysis. Total microbial genomic DNA was extracted from feces using a commercial DNA extraction kit. The V3–V4 hypervariable regions of the bacterial 16S rRNA gene were amplified using the primers 341F (5′‐ACTCCTACGGGAGGCAGCAG‐3′) and 806R (5′‐GGACTACHVGGGTATCTAAT‐3′). After quantification pf the PCR amplificons using the QuantiFluor‐ST System (Promega), high‐throughput sequencing was performed on the Illumina platform. On the basis of the above sequencing data, a suite of in‐depth statistical and visualization analyses was conducted, including multivariate analysis and differential significance tests, to characterize the community composition and phylogenetic information across multiple samples.

### 4.11 Metagenomic Sequencing

Total microbial genomic DNA was extracted from mouse fecal samples using the DNeasy PowerSoil Kit (QIAGEN, Valencia, CA, USA). The concentration and quality of the extracted DNA were assessed using spectrophotometry and agarose gel electrophoresis. Metagenomic libraries were constructed with the Illumina TruSeq Nano DNA LT Library Preparation Kit and subsequently sequenced on the DNBSEQ platform (BGI, China). Raw sequencing reads were processed to remove low‐quality reads, reads with excessive N bases, and adapter sequences using SOAPnuke (v1.5.0). Sequencing reads were aligned against the host genome using Bowtie2 (v2.2.5) to eliminate host genomic fragments. The resulting high‐quality clean data were *de novo* assembled for each sample. Scaffolds longer than 300 bp were assembled using MEGAHIT (v1.1.3). Gene prediction on the assembled contigs was performed with MetaGeneMark (v3.38). To profile the taxonomic composition, scaffold sequences were aligned against the NCBI bacterial reference database using BLASTN (E‐value < 0.001). The relative abundance of taxa at each level (phylum to species) was calculated based on the alignment results. Wilcoxon rank‐sum test and Kruskal–Wallis's test was employed for statistical significance analysis of differential abundances. Functional annotation of predicted genes was carried out by aligning them against the CAZy database for carbohydrate‐active enzymes and other relevant databases (e.g., KEGG, eggNOG).

### 4.12 RNA Sequencing

RNA was extracted from hippocampal tissue or cell samples using the TRIzol reagent (Invitrogen, Carlsbad, CA, USA). The RNA concentration was assessed using a Nanodrop Spectrophotometer (Thermo Scientific, USA). High‐quality RNA was used for cDNA library construction and subsequently sequenced on the Illumina NovaSeq X Plus platform. Differential gene expression analysis between groups was performed using the DESeq2 package. Genes with a false discovery rate (FDR) < 0.05 and an absolute log_2_ fold change ≥ 1 were considered significantly differentially expressed. Functional enrichment analysis was conducted on the set of differentially expressed genes. Gene Ontology (GO) term enrichment analysis was performed using the GO database (http://geneontology.org/). Pathway enrichment analysis was carried out using the Kyoto Encyclopedia of Genes and Genomes (KEGG) database (https://www.genome.jp/kegg/) and the Reactome database. Additionally, Gene Set Enrichment Analysis (GSEA) was performed using the Signal2Noise metric to identify pathways and biological processes enriched in a coordinated manner across the entire expression dataset.

### 4.13 RT‐qPCR

Total RNA was extracted using the RNA extraction kit (Accurate Biotechnology, Hunan, China). Subsequently, cDNA was synthesized via reverse transcription using the Evo M‐MLV RT Kit (Accurate Biotechnology, Hunan, China). Quantitative PCR was performed using the SYBR Green Premix Pro Taq HS qPCR Kit (Accurate Biotechnology, Hunan, China). The expression levels of target genes were normalized to that of the *Gapdh* housekeeping gene, and the relative gene expression was calculated using the 2^−ΔΔCt^ method. Specific primer sequences were listed in Table S6.

### 4.14 Western Blot Analysis

Total protein was extracted from the samples using a commercial protein extraction reagent. The sample proteins were separated by SDS‐PAGE and subsequently wet‐transferred onto polyvinylidene difluoride (PVDF) membranes (Millipore, Billerica, MA, USA). Immunodetection was performed using the appropriate primary and secondary antibodies, and visualization was achieved with enhanced chemiluminescence (ECL) reagents (Millipore Corporation, Billerica, MA, USA). Protein bands were quantified using ImageJ software (ImageJ 1.4, NIH, USA).

### 4.15 H&E and Immunofluorescence Staining

Following euthanasia, mouse brain tissue was immediately dissected and fixed in 4% (v/v) paraformaldehyde, and subsequently embedded in paraffin. Tissue sections were subjected to hematoxylin and eosin (H&E) staining and immunofluorescence staining. Stained sections were observed under a light microscope (Olympus, Tokyo, Japan) and analyzed using ImageJ software. For neuron dendrite staining in primary hippocampal neurons, the β3‐Tubulin antibody was used. After fixation, washing, permeabilization, and blocking, they were then incubated overnight at 4°C with primary antibodies. After washing, samples were incubated with secondary goat anti‐rabbit IgG H&L antibody (Alexa Fluor 488) for 1 h at room temperature in the dark. Cell nuclei were counterstained with DAPI, and images were acquired using a laser scanning confocal microscope (Zeiss LSM 980, Germany).

### 4.16 Transmission Electron Microscopy (TEM) Staining and Golgi Staining

Following euthanasia, the hippocampus was rapidly dissected from the mouse brains, and immediately immersed in TEM fixative for preservation. After dehydration, embedding, sectioning, and staining, the samples were observed under a TEM (HT7800, Japan) at an accelerating voltage of 80 kV. Golgi staining was performed using the FD Rapid GolgiStain Kit (FD NeuroTechnologies, USA). The stained sections were visualized under a light microscope (Revolution‐XD, Andorra, UK) and the neuronal dendrites were analyzed using the Simple Neurite Tracer (SNT) and Sholl analysis plugins integrated in ImageJ software.

### 4.17 Composition Analysis of QF and GQF

The chemical composition of QF and GQF was profiled using an SCIEX QTRAP 6500+ triple quadrupole mass spectrometer equipped with an IonDrive Turbo V electrospray ionization (ESI) source. Chromatographic separation was achieved on an ACQUITY UPLC HSS T3 column (1.8 µm, 2.1 × 100 mm; Waters) maintained at 40°C. Briefly, 50 mg of GQF was weighed into a 1.5 mL centrifuge tube. A 700 µL aliquot of a pre‐cooled (−40°C) methanol/water mixture (3:1, v/v) containing the internal standard 2‐chlorophenylalanine was added. The mixture was vortexed vigorously for 30 s and then homogenized using a homogenizer at 40 Hz for 4 min, followed by sonication in an ice‐water bath for 5 min. This homogenization‐sonication cycle was repeated three times. The sample was subsequently incubated overnight at 4°C on a thermomixer. After centrifugation at 12,000 rpm for 15 min at 4°C, the supernatant was collected, filtered through a 0.22 µm membrane, diluted five‐fold with methanol/water (3:1, v/v), vortexed for 30 s, and transferred to a vial for analysis. Mass spectrometric detection was performed in multiple reaction monitoring (MRM) mode. The mobile phase consisted of (A) 0.1% formic acid in water and (B) acetonitrile. The gradient elution program was as follows: 0–0.5 min, 2% B; 0.5–10 min, 2–50% B; 10–11 min, 50–95% B; 11–13 min, 95% B; 13–13.1 min, 95–2% B; 13.1–15 min, 2% B. The flow rate was 0.4 mL/min, and the injection volume was 2 µL with the autosampler maintained at 4°C. The ESI source parameters were set as follows: ion spray voltage, +5500/−4500 V; curtain gas, 35 psi; source temperature, 400°C; nebulizer gas (Gas 1) and auxiliary gas (Gas 2), 60 psi each; declustering potential, ±100 V. The relative content of each component was calculated as the ratio of the individual peak area to the total peak area of all detected components in the same sample.

### 4.18 Identification of Polyphenol Profiles in QF and GQF

The polyphenol profiles of QF and GQF were analyzed using a UHPLC‐Q‐Exactive Orbitrap‐MS system (Thermo Scientific, USA). Separation was performed on a Hypersil GOLD aQ column (100 × 2.1 mm; Thermo Scientific) maintained at 25°C. Extraction of free and bound polyphenols was performed. For free polyphenols, 1 g of sample was extracted twice with 25 mL of 80% acetone under ultrasonication (500 W) at room temperature for 30 min per extraction. The combined extracts were centrifuged at 3000 rpm for 25 min at room temperature. The supernatant was collected, concentrated under reduced pressure at 45°C, reconstituted in 10 mL methanol, and filtered through a 0.45 µm organic membrane. The residue from the free polyphenol extraction was used for bound polyphenol analysis. It was defatted by mixing with 20 mL of *n*‐hexane, vortexing, and centrifuging at 3000 rpm for 5 min. The defatted residue was then hydrolyzed with 17 mL of 11% methanol‐HCl solution (v/v) at 70°C for 1 h. After hydrolysis, the mixture was extracted twice with ethyl acetate. The combined organic phases were centrifuged, concentrated, and filtered under same conditions. The mobile phase consisted of (A) 0.9% acetic acid in water and (B) methanol. The gradient elution program was: 0–9 min, 20–100% B; 9–10 min, 100% B; 10–11 min, 100–20% B; 11–14 min, 20% B. The flow rate was 0.3 mL/min, and the injection volume was 10 µL. Mass detection was performed in full MS scan mode from 100 to 850 m/z. The ESI source was operated in both positive and negative ion modes with a spray voltage of 2.8 kV. The capillary and heater temperatures were set to 300°C. Sheath gas and auxiliary gas (both N_2_) flows were maintained at 40 and 10 units/min, respectively. Individual polyphenols were quantified using standard curves constructed from serial dilutions of authentic standards. Results are expressed as µg of compound per g of dry sample weight.

### 4.19 Determination of Ferulic Acid Esterase (FAE) Activity

*R. hominis* and *R. intestinalis* strains were routinely cultured and stabilized in an anaerobic chamber at 37°C, respectively. FAE activity in the culture supernatants and in mouse feces was determined using an enzyme‐linked immunosorbent assay (ELISA) kit, following the manufacturer's protocol.

### 4.20 Ex Vivo Fermentation Experiments

To determine whether *Roseburia* promotes the release of FA from GQF, an in vitro co‐culture assay was performed. *R. hominis* and *R. intestinalis* were cultured anaerobically at 37°C until the late exponential phase. For each strain, 50 mg of irradiation‐sterilized (10 kGy electron beam) GQF was added to 10 mL of fresh bacterial culture. Fresh bacterial culture without GQF served as the control, and all groups were incubated anaerobically at 37°C for 72 h without shaking.

To investigate the effect of *Roseburia* on FA release from GQF, a fecal microbiota fermentation assay was conducted. After the intervention, fresh feces were collected from mice in the AHFD and ARH groups, respectively, and immediately resuspended in PBS to a concentration of 0.125 g/mL. Then, 50 mg of irradiation‐sterilized (10 kGy electron beam) GQF was added to each fecal suspension, while PBS alone with GQF served as the control. All groups were anaerobically incubated for 72 h without shaking.

All procedures were performed aseptically, and all experiments were conducted in triplicate. After incubation, FA levels in the fermentation supernatants were quantified using a targeted Triple Quad 5500+ QTRAP system. A 200 µL of supernatant was mixed with 4 mL of chromatographic‐grade methanol. The mixture was homogenized using a high‐speed disperser for 4 min and allowed to extract at room temperature for 10 min. After brief vortexing, samples were centrifuged at 12,000 × g for 10 min at 4°C. A 0.5 mL aliquot of the supernatant was collected, vacuum‐concentrated to dryness at room temperature, reconstituted in acetonitrile, filtered through a 0.22 µm organic membrane, and transferred to a sample vial for instrumental analysis. Gradient elution was performed using mobile phase A (1.0% acetic acid in water) and mobile phase B (acetonitrile). The column temperature was maintained at 30°C, the flow rate was 0.3 mL/min, and the injection volume was 20 µL. FA was detected and quantified in Selected Ion Monitoring (SIM) mode using an external calibration curve prepared from an authentic FA standard.

### 4.21 Tissue Distribution of Ferulic Acid

FA levels in mouse cecum, liver, brain, serum, feces, and cecal contents were quantified using a targeted Triple Quad 5500+ QTRAP system. All samples were maintained on liquid nitrogen throughout the extraction procedure. For tissue samples, 50 mg of tissue was weighed and immediately transferred to a 2 mL homogenization tube containing grinding beads, followed by the rapid addition of 700 µL of chromatographic‐grade methanol. After homogenization using a tissue homogenizer, the mixture was incubated at room temperature for 10 min to facilitate extraction, vortexed briefly, and then centrifuged at 12 000 × g for 10 min at 4°C. The supernatant was collected, vacuum‐concentrated to dryness at room temperature, reconstituted in acetonitrile, filtered through a 0.22 µm organic membrane, and transferred to a sample vial for instrumental analysis. For fecal and cecal content samples, 100 mg of mouse feces and 50 mg of cecal contents were weighed, respectively. Samples were processed identically to the tissue protocol described above, beginning with transfer to a bead‐beating tube containing methanol. For the serum sample, 40 µL of serum was transferred to a 1.5 mL centrifuge tube, mixed with 1 mL of 40% (v/v) methanol‐water solution, and then spiked with 5 µL of 100 nM citric acid. The mixture was vortexed, incubated at room temperature for 10 min, and then centrifuged at 12 000 × g for 10 min at 4°C. The supernatant was collected, vacuum‐concentrated to dryness at room temperature, reconstituted in 10% acetonitrile (v/v in water), filtered through a 0.22 µm membrane, and transferred to a sample vial for instrumental analysis.

### 4.22 Statistical Analysis

Statistical analyses were performed using GraphPad Prism 9.0. The Shapiro–Wilk test was performed to evaluate the normal distribution of data, and the Brown–Forsythe test was used for homogeneity of variance assessment. Normally distributed data with homogeneous variance were analyzed by one‐way ANOVA followed by Tukey's post hoc multiple comparison test. Welch's corrected one‐way ANOVA was applied when the homogeneity of variance was not satisfied. For analyzing differences in microbial community composition or abundance data, non‐parametric tests were used: the Wilcoxon rank‐sum test for comparisons between two groups and the Kruskal‐Wallis test with Dunn's correction for comparisons among more than two groups. The relationships between FA levels, specific microbial taxa, and cognitive performance metrics were assessed using Spearman's correlation analysis. A p‐value < 0.05 was considered statistically significant.

## Author Contributions

X.L. conceptualized and supervised the study. Y.L. designed the experiments and wrote the initial draft of the manuscript. Y.L., W.Z., and L.W. performed the bioinformatic and data analysis. Y.L., S.Z., Y.S., and X.W. conducted the experiments. Y.W. and X.Y. offered help in the sequencing experiment and composition analysis. Y.L., S.M, J.G., R.G., Y.W., and X.Y. were responsible for visualization. X.L. reviewed and edited the manuscript.

## Funding

This research was financially supported by the National Key Research and Development Program of China (No. K3010325101), National Key Research and Development Program of China (No. 2025YFF1107600), and Laboratory for Research and Utilization of Qinghai Tibet Plateau Germplasm Resources Project (2025).

## Conflicts of Interest

The authors declare no conflicts of interest.

## Supporting information




**Supporting File 1**: advs76686‐sup‐0001‐SuppMat.docx.

**Supporting File 2**: advs76686‐sup‐0002‐TableS1‐S6.docx.

## Data Availability

Metagenomic and 16S rRNA sequencing data in this study are available in the NCBI Sequence Read Archive (SRA) under project numbers PRJNA1442109 and PRJNA1441750. The RNA‑seq data of mouse tissues and primary hippocampal neuron cells are available in NCBI SRA database under project numbers PRJNA1442833 and PRJNA1442313. Metabolomics data are deposited to the EMBL‐EBI MetaboLights database with MetaboLights number MTBLS14185.
