# Supplementary material for: Gut Microbial Release of Ferulic Acid From Germinated Quinoa Alleviates Obesity‐Associated Cognitive Impairment by Activating Hippocampal Mitophagy Associated with PINK1/Parkin Pathway
Source: Adv Sci (Weinh). 2026 Jul 20;13(57):e76686. doi: 10.1002/advs.76686 (PMC13383685; doi:10.1002/advs.76686)
Supplement: Supplementary file 1 [file ADVS-13-e76686-s001.docx]

**Gut microbial release of ferulic acid from germinated quinoa alleviates obesity-associated cognitive impairment by activating hippocampal mitophagy** **associated with PINK1/Parkin pathway**

Yongli Lan^1^, Wengang Zhang^2^, Lei Wang^3^, Shiyang Zhao^1^, Yujie Song^1^, Xinze Wang^1^, Yutang Wang^1^, Xijuan Yang^2^, Shaobo Ma^1^, Junlin Ge^1^, Rui Guo^1^, Xuebo Liu^1,*^

*^1^College of Food Science and Engineering, Northwest A&F University, No. 22 Xinong Road, Yangling, Shaanxi 712100, China*

*^2^Academy of Agriculture and Forestry Sciences, Laboratory for Research and Utilization of Qinghai Tibet Plateau Germplasm Resources, Qinghai University, Xining 810016, China*

*^3^Department of Clinical Nutrition, Xijing Hospital, Air Force Military Medical University, Xi’an 710000, China*

*Corresponding author:

Xuebo Liu, College of Food Science and Engineering, Northwest A&F University, No. 22 Xinong Road, Yangling, Shaanxi, China 712100. *E-mail: xuebofood@nwafu.edu.cn

Table S1 Relative content of key differential metabolites screened based on fold change value

| Compounds | KEGG_ID | Relative content (%) | | FC | down/up |
| --- | --- | --- | --- | --- | --- |
|  |  | QF | GQF |  |  |
| 3,4-Dihydroxymandelic acid | C05580 | 0.003±0.000 | 0.003±0.001 | 0.861 | down |
| Glucose 1-phosphate | C00103 | 0.035±0.005 | 0.013±0.004 | 0.358 | down |
| 4-Hydroxyphenylacetylglutamic acid | C05595 | 0.492±0.078 | 0.163±0.050 | 0.330 | down |
| Avenanthramide A | C08472 | 0.001±0.000 | 0.000±0.000 | 0.317 | down |
| Dihydrocapsaicin | C16952 | 0.003±0.001 | 0.001±0.000 | 0.281 | down |
| D-Aspartic acid | C00402 | 0.041±0.006 | 0.009±0.001 | 0.216 | down |
| L-Aspartic acid | C00049 | 0.041±0.006 | 0.009±0.001 | 0.213 | down |
| 5,7-Dihydroxyisoflavone | C02168 | 0.000±0.000 | 0.000±0.000 | 0.180 | down |
| Lincomycin | C06812 | 0.002±0.001 | 0.000±0.000 | 0.170 | down |
| Pinocembrin | C09827 | 0.001±0.001 | 0.050±0.009 | 46.399 | up |
| 5'-S-Methyl-5'-thioadenosine | C00170 | 0.031±0.004 | 1.174±0.178 | 37.432 | up |
| Uridine 5'-diphospho-D-glucose; UDP-D-galactose | C00029; C00052 | 0.000±0.000 | 0.010±0.002 | 32.811 | up |
| D-glucoronic acid | C00191 | 0.064±0.007 | 1.419±0.233 | 22.330 | up |
| beta-Nicotinamide mononucleotide | C00455 | 0.002±0.000 | 0.018±0.003 | 11.596 | up |
| Uncinatone | C09976 | 0.001±0.000 | 0.006±0.000 | 10.799 | up |
| 4-O-alpha-D-Galactopyranuronosyl-D-galacturonic acid | C02273 | 0.001±0.000 | 0.010±0.002 | 11.410 | up |
| Asp-Phe methyl ester; Aspartame | C11045 | 0.007±0.001 | 0.070±0.012 | 10.294 | up |
| Ferulic acid | C01494 | 0.029±0.007 | 0.273±0.028 | 9.272 | up |
| Beta-Aminopropionitrile | C05670 | 0.000±0.000 | 0.001±0.000 | 10.271 | up |
| Riboflavine | C00255 | 0.005±0.002 | 0.044±0.004 | 8.880 | up |
| Lansiumarin A | — | 0.001±0.000 | 0.012±0.004 | 8.498 | up |
| Adenosine 2',3'-cyclic phosphate | C02353 | 0.003±0.001 | 0.027±0.005 | 8.458 | up |
| Glycitein | C14536 | 0.002±0.001 | 0.018±0.001 | 8.049 | up |
| 3-Hydroxy-4-methoxycinnamic acid; Isoferulic acid | C10470 | 0.009±0.002 | 0.069±0.013 | 7.951 | up |

Values are means ± standard deviation (SD). “—”: not found in database.

Table S2 Contents of free and bound phenolic compounds in germinated and non-germinated quinoa

| Phenolic compounds (μg/g DW) | Quinoa flour (QF) | | Germinated quinoa flour (GQF) | |
| --- | --- | --- | --- | --- |
|  | Free | Bound | Free | Bound |
| Ferulic acid | 147.29±2.10 | 419.45±3.73 | 328.32±8.77 | 971.04±2.25 |
| Catechin hydrate | 0.33±0.02 | 2.69±0.02 | 4.23±0.17 | 3.35±0.17 |
| Chlorgenic acid | 0.61±0.02 | ND | 25.66±0.95 | ND |
| Cianidanol | 0.29±0.10 | 3.01±0.21 | 3.79±0.15 | 3.55±0.05 |
| 4-hydroxybenzoic acid | 43.50±0.27 | 32.22±0.23 | 105.65±2.68 | 203.64±3.94 |
| 2,4-dihydroxybenzoic acid | 1.16±0.09 | 8.70±0.11 | 1.77±0.03 | 6.65±0.14 |
| Epicatechin | ND | 0.14±0.04 | 0.18±0.04 | 2.03±0.02 |
| Syringic acid | 5.66±0.03 | 43.85±0.55 | 9.95±0.32 | 6.95±0.08 |
| Maltol | 12.59±2.58 | 72.52±2.49 | 14.33±0.59 | 35.59±0.65 |
| Naringin | 1.97±0.06 | 1.93±0.01 | 2.47±0.02 | 3.13±0.10 |
| Homoorientin | 0.72±0.06 | 11.86±0.97 | 0.77±0.01 | 26.07±0.11 |
| Isoferulic acid | 10.68±2.01 | 130.45±8.41 | 40.73±2.78 | 311.51±7.36 |
| 4-Hydrosxybenzaldehyde | 22.04±1.49 | 64.74±2.81 | 26.39±1.40 | 88.28±4.96 |
| Caffeic acid | 22.82±0.12 | 25.31±0.14 | 130.07±6.25 | 281.16±2.42 |
| trans-4-hydroxycinamic acid | 111.97±4.94 | 253.63±4.12 | 278.67±2.03 | 534.10±8.34 |
| Vanillic acid | 122.58±2.15 | 697.82±6.24 | 164.15±2.87 | 210.15±1.45 |
| Isovitexin | 0.40±0.02 | 0.25±0.02 | 3.55±0.17 | 0.13±0.03 |
| Gallic acid | 7.03±0.07 | 20.91±0.29 | 8.29±0.13 | 23.17±0.19 |
| Kaempferol | 4.39±0.12 | 0.56±0.01 | 2.20±0.07 | 169.75±1.29 |
| Luteolin | 3.18±0.03 | 0.69±0.01 | 7.99±0.04 | 11.21±0.03 |
| Hesperidin | ND | ND | 0.53±0.01 | ND |
| Psoralidin | 0.16±0.01 | 0.13±0.03 | 0.46±0.01 | 20.09±2.01 |
| 2-hydroxycinnamic acid | 20.78±3.30 | 797.37±9.05 | 19.63±0.78 | 140.06±3.88 |
| 6-Gingerol | ND | ND | 2.93±0.06 | ND |
| Benzoic acid | 30.08±2.30 | 140.69±6.12 | 16.62±1.44 | 286.76±7.06 |
| Naringenin | 6.81±0.03 | 2.40±0.01 | 20.94±0.78 | 22.87±3.06 |
| Kaempferol-3-o-rutinoside | 803.12±1. 39 | ND | 738.53±3.77 | ND |

Table S2 (Continued)

| Phenolic compounds (μg/g DW) | Quinoa flour (QF) | | Germinated quinoa flour (GQF) | |
| --- | --- | --- | --- | --- |
|  | Free | Bound | Free | Bound |
| Isoquercitrin | 349.29±1.91 | ND | 177.35±0.79 | 60±4.01 |
| Quercetin | 1.12±0.03 | 0.40±0.01 | 3.53±0.02 | 22.77±0.13 |
| Diosmetin | 11.60±0.28 | 35.68±4.07 | 146.39±1.48 | 45.47±0.04 |
| Myricetin | 0.18±0.01 | 0.55±0.01 | 0.10±0.01 | 10.19±0.02 |
| Diosmin | 27.45±2.10 | ND | 90.65±4.57 | ND |
| Rutin | 960.91±3.94 | 13.94±1.03 | 359.21±5.43 | 117.43±2.44 |
| Vitexin | 3.34±0.10 | ND | 0.86±0.09 | ND |

Values are means ± standard deviation (SD). ND: not detected.

Table S3 Nutritional components in germinated and non-germinated quinoa

| Sample | Nutritional components (mg/100 g) | | | | | |
| --- | --- | --- | --- | --- | --- | --- |
|  | Protein | Fat | Carbohydrate | Moisture | Ash | Fiber |
| Quinoa Flour | 12.4±0.63 | 5.4±0.11 | 65.7±0.15 | 8.45±0.08 | 2.1±0.02 | 5.88±0.09 |
| Germinated quinia flour | 14.1±0.18 | 5.2±0.07 | 62.1±0.01 | 6.39±0.03 | 2.1±0.01 | 10.1±0.07 |

Table S4 Diet composition

| Product | Groups | | | | | |
| --- | --- | --- | --- | --- | --- | --- |
|  | CON (D12450J) | | HFD (D12492) | | LQF (10% quinoa flour) | |
|  | gm% | kcal% | gm% | kcal% | gm% | kcal% |
| Protein | 19.2 | 20.0 | 26.2 | 20.0 | 25.9 | 20.0 |
| Carbohydrate | 67.3 | 70.0 | 26.3 | 20.1 | 24.7 | 19.1 |
| Fat | 4.3 | 10.0 | 34.9 | 59.9 | 34.6 | 59.9 |
| Total |  | 100.0 |  | 100.0 |  | 99.0 |
| kcal/gm | 3.85 |  | 5.24 |  | 5.19 |  |
| Ingredient | gm | kcal | gm | kcal | gm | kcal |
| Casein | 200 | 800 | 200 | 800 | 190 | 761.312 |
| L-Cystine | 3 | 12 | 3 | 12 | 3 | 12 |
| Corn Starch | 506.2 | 2024.8 | 0 | 0 | 0 | 0 |
| Maltodextrin 10 | 125 | 500 | 125 | 500 | 125 | 500 |
| Sucrose | 68.8 | 275.2 | 68.8 | 275.2 | 17 | 68 |
| Quinoa Flour | 0 | 0 | 0 | 0 | 78 | 281.58 |
| Germinated Quinoa Flour | 0 | 0 | 0 | 0 | 0 | 0 |
| Cellulose | 50 | 0 | 50 | 0 | 45 | 0 |
| Soybean Oil | 25 | 225 | 25 | 225 | 25 | 225 |
| Lard | 20 | 180 | 245 | 2205 | 241 | 2169 |
| Mineral Mix | 10 | 0 | 10 | 0 | 10 | 0 |
| DiCalcium Phosphate | 13 | 0 | 13 | 0 | 13 | 0 |
| Calcium Carbonate | 5.5 | 0 | 5.5 | 0 | 5.5 | 0 |
| Potassium Citrate | 16.5 | 0 | 16.5 | 0 | 16.5 | 0 |
| Vitamin Mix | 10 | 40 | 10 | 40 | 10 | 40 |
| Choline Bitartrate | 2 | 0 | 2 | 0 | 2 | 0 |

Table S5 Diet composition

| Product | Groups | | | | | |
| --- | --- | --- | --- | --- | --- | --- |
|  | HQF (30% quinoa flour) | | LGQF (10% germinated quinoa flour) | | HGQF (30% germinated quinoa flour) | |
|  | gm% | kcal% | gm% | kcal% | gm% | kcal% |
| Protein | 25.9 | 20.0 | 26.0 | 20.0 | 25.9 | 20.0 |
| Carbohydrate | 24.8 | 19.1 | 24.8 | 19.1 | 24.7 | 19.0 |
| Fat | 34.6 | 59.9 | 34.6 | 59.9 | 34.6 | 60.0 |
| Total |  | 99.0 |  | 99.0 |  | 99.0 |
| kcal/gm | 5.08 |  | 5.20 |  | 5.12 |  |
| Ingredient | gm | kcal | gm | kcal | gm | kcal |
| Casein | 170 | 681.456 | 189 | 756.008 | 166 | 665.768 |
| L-Cystine | 3 | 12 | 3 | 12 | 3 | 12 |
| Corn Starch | 0 | 0 | 0 | 0 | 0 | 0 |
| Maltodextrin 10 | 37 | 148 | 125 | 500 | 45 | 180 |
| Sucrose | 0 | 0 | 20 | 80 | 0 | 0 |
| Quinoa Flour | 239 | 862.79 | 0 | 0 | 0 | 0 |
| Germinated Quinoa Flour | 0 | 0 | 78 | 274.248 | 238 | 836.808 |
| Cellulose | 36 | 0 | 42 | 0 | 26 | 0 |
| Soybean Oil | 25 | 225 | 25 | 225 | 25 | 225 |
| Lard | 232 | 2088 | 241 | 2169 | 233 | 2097 |
| Mineral Mix | 10 | 0 | 10 | 0 | 10 | 0 |
| DiCalcium Phosphate | 13 | 0 | 13 | 0 | 13 | 0 |
| Calcium Carbonate | 5.5 | 0 | 5.5 | 0 | 5.5 | 0 |
| Potassium Citrate | 16.5 | 0 | 16.5 | 0 | 16.5 | 0 |
| Vitamin Mix | 10 | 40 | 10 | 40 | 10 | 40 |
| Choline Bitartrate | 2 | 0 | 2 | 0 | 2 | 0 |

Table S6 Primer sequences used for quantitative RT-qPCR analysis

|  | Forward Primer | Reverse Primer |
| --- | --- | --- |
| *Bdnf* | CTGGATGAGGACCAGAAG | CCTCCAGCAGAAAGAGTAG |
| *Ngf* | CGACTCCAAACACTGGAACTCA | GCCTGCTTCTCATCTGTTGTCA |
| *Nt3* | GTTCCAGCCAATGATTGCAA | GGGCGAATTGTAGCGTCTCT |
| *Nt4* | GAGGCACTGGCTCTCAGAATG | CGAATCCAGCGCCAGC |
| *Psd-95* | TCTGTGCGAGAGGTAGCAGA | AAGCACTCCGTGAACTCCTG |
| *Syp* | GTGCCAACAAGACGGAGAGTGC | CAGCCGAGGAGGAGTAGTCACC |
| *16S* | AGAGTTTGATCCTGGCTCAG | CTGCTGCCTCCCGTAGGAGT |
| *Fae* | ACGGCTGAGACGGTGGAAGTG | CGGCTGGGATCTTTCTGGCTTC |
| *R.hominis* | GCTTCCGTGCGTGCTTGT | CCGTACTTCACGGCAAGCTT |
| *Gapdh* | CCAGGTTGTCTCCTGCGACTT | CCTGTTGCTGTAGCCGTATTCA |
