# Supplementary material for: Gut Microbial Release of Ferulic Acid From Germinated Quinoa Alleviates Obesity‐Associated Cognitive Impairment by Activating Hippocampal Mitophagy Associated with PINK1/Parkin Pathway
Source: Adv Sci (Weinh). 2026 Jul 20;13(57):e76686. doi: 10.1002/advs.76686 (PMC13383685; doi:10.1002/advs.76686)
Supplement: Supplementary file 2 [file ADVS-13-e76686-s002.docx]

**Gut microbial release of ferulic acid from germinated quinoa alleviates obesity-associated cognitive impairment by activating hippocampal mitophagy** **associated with PINK1/Parkin pathway**

Yongli Lan^1^, Wengang Zhang^2^, Lei Wang^3^, Shiyang Zhao^1^, Yujie Song^1^, Xinze Wang^1^, Yutang Wang^1^, Xijuan Yang^2^, Shaobo Ma^1^, Junlin Ge^1^, Rui Guo^1^, Xuebo Liu^1,*^

*^1^College of Food Science and Engineering, Northwest A&F University, No. 22 Xinong Road, Yangling, Shaanxi 712100, China*

*^2^Academy of Agriculture and Forestry Sciences, Laboratory for Research and Utilization of Qinghai Tibet Plateau Germplasm Resources, Qinghai University, Xining 810016, China*

*^3^Department of Clinical Nutrition, Xijing Hospital, Air Force Military Medical University, Xi’an 710000, China*

*Corresponding author:

Xuebo Liu, College of Food Science and Engineering, Northwest A&F University, No. 22 Xinong Road, Yangling, Shaanxi, China 712100. *E-mail: xuebofood@nwafu.edu.cn

**Figure S1.** **GQF ameliorates obesity and cognitive deficits in HFD-induced mice, related to Figure 1.**

(A) Body weight change during intervention (n = 10). (B) Body weight gain (n = 10). (C) Food intake (n = 10). (D) Water intake (n = 10). (E) Energy intake (n = 10). (F) Representative H&E staining diagram of subcutaneous white adipose tissue (sWAT), epididymal white adipose tissue (eWAT), and brown white adipose tissue (BAT) (n = 3). Scale bar, 100 μm. (G−I) Weight of sWAT, eWAT, and BAT (n = 10). (J) Representative H&E staining diagram of hippocampal neuron morphology (n = 3). Scale bar, 100 μm. The arrowheads stand for the representative neuron. (K−N) Relative levels of *Bdnf*, *Ngf*, *Nt3*, and *Nt4* mRNA in hippocampus (n = 6). (O) Representative immunofluorescence staining images of BDNF from hippocampal sections. Scale bar, 100 μm. The arrowheads stand for the representative BDNF. (P) Quantification of the BDNF area based on immunofluorescence staining sections by ImageJ software in the hippocampus (n = 9 slices from 3 mice). (Q−R) Relative levels of *Psd-95* and *Syp* mRNA in hippocampus (n = 6). Data are presented as the mean ± SEM. **p* < 0.05, ***p* < 0.01, and ****p* < 0.001 vs. CON group. #*p* < 0.05, ##*p* < 0.01, and ###*p* < 0.001 vs. HFD group.

**Figure S2. GQF alters the gut microbiota composition in HFD-induced obese mice, related to Figure 2.**

(A) Plot of species accumulation curves. (B) OUT Venn diagram. (C) Averaged relative abundance of bacteria at the genus level. (D) Linear discriminant Effect Size (LEfSe) analysis. (E−G) Pairwise comparisons of gut microbial taxa abundance between experimental groups of *Butyricicoccus* (E), *Ruminococcus* (F), and *Odoribacter* (G) at the genus level. Non-parametric comparisons across four groups were performed using the Kruskal-Wallis H test. For taxa with a significant overall effect (*p* < 0.05), post-hoc pairwise comparisons were conducted using Dunn’s test with the Benjamini-Hochberg FDR correction to control for multiple comparisons. n = 8 mice per group.

**Figure S3.** **Effect of fecal microbiota transplantation on gut microbiota and cognitive impairment in HFD-induced obese mice, related to Figure 3.**

(A−B) Linear discriminant analysis (LDA) of microbial composition based on differentially expressed taxa (LDA scores > 2.0 and adjusted *p* < 0.05) (n = 6). (C) Body weight change during intervention (n = 8). (D) Body weight gain (n = 8). (E) Representative H&E staining diagram of subcutaneous white adipose tissue (sWAT), epididymal white adipose tissue (eWAT), and brown white adipose tissue (BAT) (n = 3). Scale bar, 100 μm. (F-H) Weight of sWAT, eWAT, and BAT (n = 8). (I) Representative H&E staining diagram of hippocampal neuron morphology (n = 3). Scale bar, 100 μm. The arrowheads stand for the representative neuron. (J−L) Relative levels of *Ngf*, *Nt3*, and *Nt4* mRNA in hippocampus (n = 6). (M−O) Protein expression levels of hippocampal PSD-95 and SYP in mice (n = 3). Data are presented as the mean ± SEM. **p* < 0.05, ***p* < 0.01, and ****p* < 0.001 vs. FCON group. #*p* < 0.05, ##*p* < 0.01, and ###*p* < 0.001 vs. FHFD group.

**Figure S4. Ferulic acid (FA) is a key mediator underlying the protective effects of GQF against cognitive impairment in obese mice, related to Figure 4.**

(A) Representative total ion chromatogram. (B) Plot of principal components analysis of QF and GQF samples (n = 3). (C−D) Pie chart of chemical composition of QF (C) and GQF (D). (E) Permutation test of OPLS-DA model for group GQF vs QF. (F) Plot of principal components analysis of fecal metagenome (n = 5). (G) Chao index. (H) Shannon index. (I) Level 1 annotated map of KEGG. (J) Annotated map of CAZy for fecal metagenome. (K) Linear discriminant Effect Size (LEfSe) analysis. (L) Linear discriminant analysis (LDA) of microbial composition based on differentially expressed taxa (LDA scores > 2.0 and adjusted *p* < 0.05). Data are presented as the median ± interquartile range. **p* < 0.05, ***p* < 0.01.

**Figure S5. GQF-induced *Roseburia* enrichment promotes FA release from GQF via bacterial feruloyl esterase (FAE), related to Figure 5.**

(A) Relative levels of *Fae* gene in mice feces of CON, HFD, HQF, and HGQF groups (n = 8). (B−C) Growth of *R. hominis* or *R. intestinalis* in medium containing FA as sole carbon source or supplemented with culture medium (n = 3 independent repeats), suggesting the lack of utilization of the FA by the bacterium. (D) After culturing *R. hominis* or *R. intestinalis* for 72 h in the medium containing FA as sole carbon source or supplemented with culture medium, the FA levels in the medium were determined (n = 3 independent repeats). (E) Protein accumulation in the medium containing FA as sole carbon source or supplemented with culture medium after culturing with *R. hominis* or *R. intestinalis* for 72 h. (n = 8 from 3 independent repeats). (F) The relative abundance of *R. hominis* in feces of pseudo germ-free mice after different treatments (n = 6). (G) Relative levels of *Fae* gene in mice feces of AHFD, AGQF, ARH, and ARG groups (n = 10). (H−N) The levels of FA were quantified in mice cecum (I), liver (J), brain (K), serum (L), feces (M), and cecal contents (N) using UPLC-MS/MS (n = 6). (O−R) Correlation analysis of entries in probe zone of Barnes maze test (O), BDNF mean fluorescence intensity (P), the width of PSD (Q), and the dendrite spine density (R) with the FA levels in mice brain. (C−E) Data are presented as the mean ± SEM. (A, F−N) Data are presented as the median ± interquartile range. **p* < 0.05, ***p* < 0.01, and ****p* < 0.001.

**Figure S6. FA ameliorates obesity and cognitive deficits in HFD-induced mice, related to Figure 6.**

(A) Body weight change during intervention (n = 10). (B) Body weight gain (n = 10). (C) Representative H&E staining diagram of subcutaneous white adipose tissue (sWAT), epididymal white adipose tissue (eWAT), and brown white adipose tissue (BAT) (n = 3). Scale bar, 100 μm. (D-F) Weight of sWAT, eWAT, and BAT (n = 10). (G) Water intake (n = 10). (H) Food intake (n = 10). (I) Energy intake (n = 10). (J) Representative H&E staining diagram of hippocampal neuron morphology (n = 3). Scale bar, 100 μm. The arrowheads stand for the representative neuron. (K−M) Relative levels of *Ngf*, *Nt3*, and *Nt4* mRNA in hippocampus (n = 6). (N−O) Protein expression levels of hippocampal BDNF in mice (n = 3). (P−Q) Representative images of Golgi staining of hippocampal pyramidal cells (P) and granule cells (Q). Scale bar, 50 μm. (R) Relative levels of *Syp* mRNA in hippocampus (n = 6). Data are presented as the mean ± SEM. **p* < 0.05, ***p* < 0.01, and ****p* < 0.001 vs. CON group. #*p* < 0.05, ##*p* < 0.01, and ###*p* < 0.001 vs. HFD group.

**Figure S7. FA mediates GQF-induced improvement of synaptic plasticity by regulating hippocampal synaptic transmission-related genes, related to Figure 7.**

(A) Plot of principal components analysis (GQF refers to the HGQF group, n = 6). (B) Volcano plot displaying 6892 differentially expressed genes (DEGs, FDR-p < 0.05) in the hippocampus from HFD vs. CON group (n = 6). *Ngfr* and other genes related to synaptic transmission and neuroplasticity are denoted. (C) GO enrichment analysis of differential biological functions in hippocampus of HFA vs. HFD group. (D−E) Representative GSEA of differential biological processes triggered by HFD. (F) Representative DiI staining images of primary hippocampal neurons treated with PA (200 μM) alone or FA pre-treatment (10, 40 μM). Scale bar, 5 μm. (G) Volcano plot displaying 2261 differentially expressed genes (DEGs, FDR-p < 0.05) in the primary hippocampal neurons from PA vs. CON group (n = 3). Genes related to synaptic transmission and neuroplasticity are denoted. (H) KEGG pathway analysis of differential biological processes in the primary hippocampal neurons from PA vs. CON group (n = 3). (I−K) Representative GSEA of differential biological processes triggered by FPA. (L−M) KEGG (L) and Reactome (M) pathway analysis of differential biological processes in hippocampus of GQF vs. HFD group (n = 6).

**Figure S8. FA and GQF promote neuronal growth and synaptic plasticity by activating hippocampal mitophagy associated with PINK1/Parkin pathway, related to Figure 8.**

(A) Representative images of hippocampal mitochondria ultrastructure by transmission electron microscope. Scale bar, 1 μm. The arrowheads stand for the representative mitophagosomes. (B) Abnormal mitochondria ratio (n = 6 slices from 3 mice). (C) Volcano plot displaying 6892 differentially expressed genes (DEGs, FDR-p < 0.05) in the hippocampus from HFD vs. CON group (n = 6). Genes related to mitophagy are denoted. (D−E) Representative confocal microscopy images (×20) and quantitative analysis showing enhanced mitophagosome accumulation in the hippocampal sections of mice treated with GQF. The density of mitophagosomes in the hippocampus was quantified and normalized to that of control mice (n = 3). (F−H) Protein expression levels of hippocampal P62 and LC3 in mice (n = 3). (I−K) Protein expression levels of hippocampal PINK1 and Parkin in mice (n = 3). Western Blots in Figure F and I were produced using one unified batch of mouse tissue samples, hence the identical GAPDH loading control bands observed between the two panels. (L) Representative immunofluorescence staining images of PINK1 and Parkin from hippocampal sections. Scale bar, 40 μm. The arrowheads stand for the representative PINK1 or Parkin. (M−N) Quantification of the PINK1 (M) and Parkin (N) area based on immunofluorescence staining sections by ImageJ software in the hippocampus (n = 6 slices from 3 mice). (O) The cell activity of SH-SY5Y cells treated with 0-600 μM PA for 12 h was determined by CCK8 method. (P) The cell activity of SH-SY5Y cells treated with 0-80 μM FA for 24 h was determined by CCK8 method. (Q) After FA (0-80 μM) pretreatment for 24 h and 300 μM PA stimulation for 12 h, the cell activity of SH-SY5Y cells was detected by CCK8 method. (R−V) After SH-SY5Y cells were treated with 40 μM FA, 25 μM Mdivi-1, 300 μM PA, 40 μM FA + 300 μM PA, or 25 μM Mdivi-1 + 40 μM FA + 300 μM PA, respectively, they were treated with fluorescence probe for staining, cells were observed by confocal microscope (×20) (R), quantitative analysis for evaluating intracellular ROS levels (S), mitochondrial membrane potential (T), Ca^2+^ levels (U) and ATP levels (V) (n = 3 independent repeats). (W−Aa) Protein expression levels of PINK1, Parkin, P62, and LC3 in 300 μM PA-treated SH-SY5Y cells at different times (n = 3). Data are presented as the means ± SEM. **p* < 0.05, ***p* < 0.01, and ****p* < 0.001 vs. CON group. (A−N) #*p* < 0.05, ##*p* < 0.01, and ###*p* < 0.001 vs. HFD group. (O−Q) ###*p* < 0.001 vs. PA group. (S−V) #*p* < 0.05, ##*p* < 0.01, and ###*p* < 0.001 vs. PA+FA group.
